# Targeting DNA polymerase to DNA double-strand breaks reduces DNA deletion size and increases templated insertions generated by CRISPR/Cas9

**DOI:** 10.1093/nar/gkac186

**Published:** 2022-03-22

**Authors:** Kyung W Yoo, Manish Kumar Yadav, Qianqian Song, Anthony Atala, Baisong Lu

**Affiliations:** Wake Forest Institute for Regenerative Medicine, Wake Forest University Health Sciences, Winston-Salem, NC 27101, USA; Wake Forest Institute for Regenerative Medicine, Wake Forest University Health Sciences, Winston-Salem, NC 27101, USA; Department of Cancer Biology, Wake Forest University Health Sciences, Winston-Salem, NC 27157, USA; Wake Forest Institute for Regenerative Medicine, Wake Forest University Health Sciences, Winston-Salem, NC 27101, USA; Wake Forest Institute for Regenerative Medicine, Wake Forest University Health Sciences, Winston-Salem, NC 27101, USA

## Abstract

Most insertions or deletions generated by CRISPR/Cas9 (clustered regularly interspaced short palindromic repeats/CRISPR-associated protein 9) endonucleases are short (<25 bp), but unpredictable on-target long DNA deletions (>500 bp) can be observed. The possibility of generating long on-target DNA deletions poses safety risks to somatic genome editing and makes the outcomes of genome editing less predictable. Methods for generating refined mutations are desirable but currently unavailable. Here, we show that fusing *Escherichia coli* DNA polymerase I or the Klenow fragment to Cas9 greatly increases the frequencies of 1-bp deletions and decreases >1-bp deletions or insertions. Importantly, doing so also greatly decreases the generation of long deletions, including those >2 kb. In addition, templated insertions (the insertion of the nucleotide 4 nt upstream of the protospacer adjacent motif) were increased relative to other insertions. Counteracting DNA resection was one of the mechanisms perturbing deletion sizes. Targeting DNA polymerase to double-strand breaks did not increase off-targets or base substitution rates around the cleavage sites, yet increased editing efficiency in primary cells. Our strategy makes it possible to generate refined DNA mutations for improved safety without sacrificing efficiency of genome editing.

## INTRODUCTION

The CRISPR/Cas9 (clustered regularly interspaced short palindromic repeats/CRISPR-associated protein 9) system uses a single effector protein to make DNA double-strand breaks (DSBs) guided by single-guide RNA (sgRNA) (1) and has been used to make specific genetic changes in human cells (2–5). *Streptococcus pyogenes* Cas9 (SpCas9) mainly generates blunt ends via cleaving the two strands that are 3 nt upstream of the NGG protospacer adjacent motif (PAM). It can also generate staggered ends with 1-, 2- or 3-nt 5′ overhangs via cleaving the targeting strand 3 nt and the nontargeting strand 4, 5 or 6 nt upstream of the PAM (6).

Human cells have multiple pathways to repair the DSBs created by CRISPR/Cas9 nucleases: homology-directed repair (HDR), canonical nonhomologous end joining (cNHEJ) and alternative end joining pathways, including microhomology-mediated end joining (MMEJ) and single-strand annealing (SSA) (7). In the cNHEJ pathway, the two ends of the DSBs are processed for religation without the involvement of resection or a template. Pol X family members (Pol λ, μ and β and terminal transferase) may function to fill in 5′ overhangs (8,9). This function can explain the widely observed predictable insertions or templated insertions (TISs) induced by SpCas9 (6,10–15).

The HDR, MMEJ and SSA repair pathways all depend on DNA resection to generate 3′ overhangs (7,16). DNA resection is initiated by the MRE11–RAD50–NBS1 complex and stimulated by CtIP (encoded by *RBBP8*) (17). MRE11’s endonuclease activity generates a nick 3′ to the DSB, and the 3′–5′ exonucleolytic activity generates a 3′ single-stranded DNA overhang from the nick (18–20). HDR uses long 3′ overhangs and the DNA template to faithfully repair the DSBs. Whereas MMEJ and SSA use microhomology (2–5 nt) and short homology (10–15 nt), respectively, in the two 3′ overhangs to facilitate DNA synthesis, generating DNA deletions of different sizes depending on the distances between the homologous regions (21,22). Long 3′ overhangs from excessive DNA resection can also be filled in by DNA polα and associated complexes (23). This action explains the observed small tandem duplicates at DSBs with 3′ overhangs generated by Cas9 nickases (24).

Although CRISPR/Cas9-induced mutation profiles are somewhat predictable, and small insertions and deletions (INDELs) are the dominant mutation types (6,10–14,25,26), unpredictable on-target large deletions are widely reported (21,27–30). Currently, methods for suppressing the generation of large deletions are lacking. Although genome editing methods without generating DSBs have been developed, such as base editing (31–33) and prime editing (34), many applications—including eradicating integrated provirus DNA from human cells (35) and removing disease-causing DNA repeats (36)—still involve DSB generation. Thus, a method that can generate more refined mutation profiles will improve safety of the ultimate applications.

**Figure 1.**
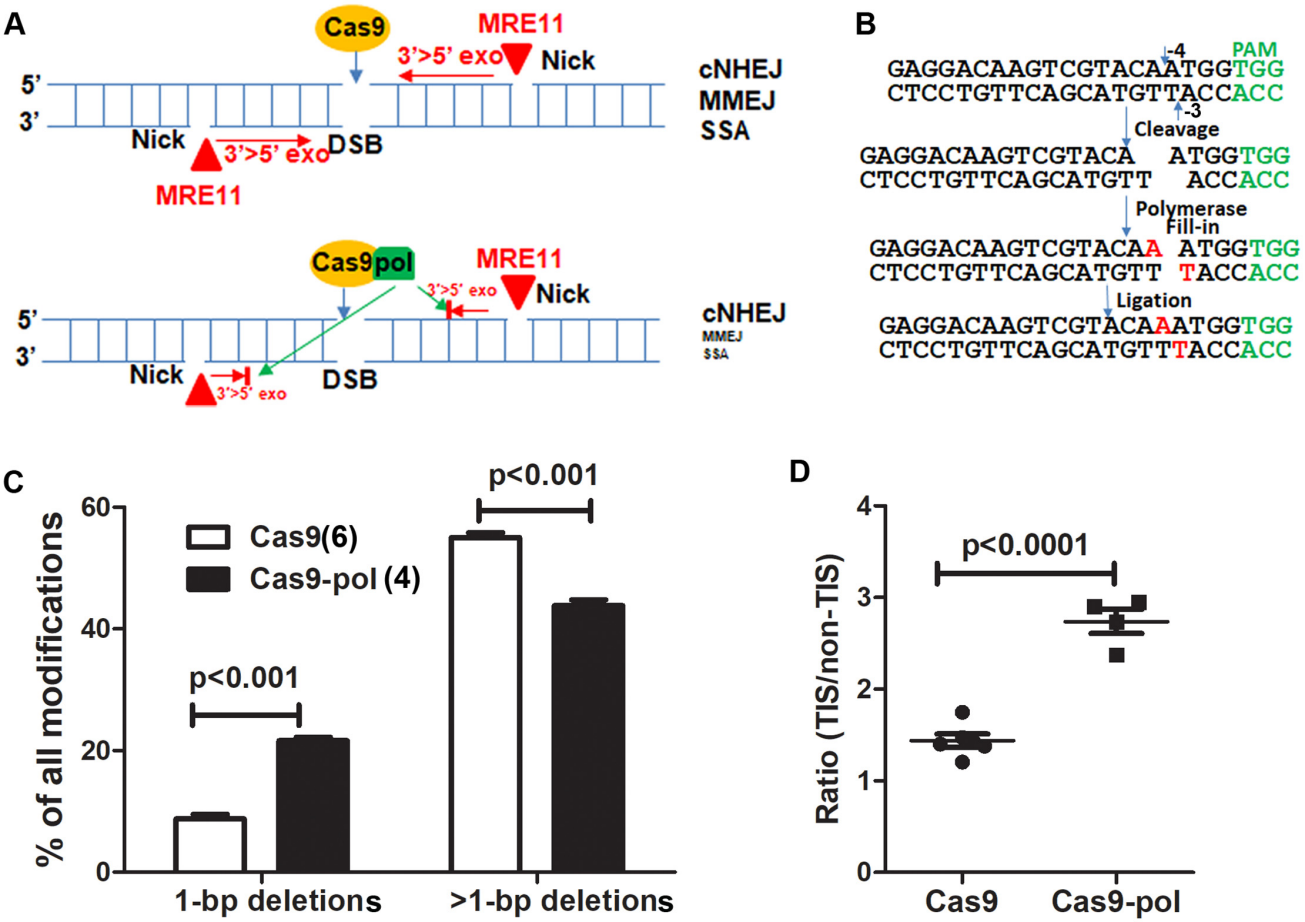
Targeting *E. coli* DNA pol I to DSBs increased the ratio of 1-bp deletions versus >1-bp deletions and TIS versus non-TIS. (**A**) Counteracting DNA resection (e.g. MRE11) by pol I fused to Cas9. The expected result is suppression of the MMEJ and SSA DNA repair pathways, which require DNA resection. (**B**) DNA polymerase generates 1-bp TIS via filling in 5′ overhangs. The red nucleotides (online version) are filled in by DNA polymerase. The target site of *CLCN5* sgRNA is used as an example. The cleavage sites on both strands to generate 1-nt 5′ overhangs are indicated by small arrows. (**C**) Fusing pol I to Cas9 increased the percentage of 1-bp deletions and decreased >1-bp deletions targeting the *CLCN5* gene in HEK293T cells. Two-way ANOVA was followed by Bonferroni post-tests. Numbers of replicates are listed in parentheses. (**D**) Fusing pol I to Cas9 increased the ratio of 1-bp TIS versus 1-bp non-TIS (two-tailed *t*-test).

*Escherichia coli* DNA polymerase I (pol I) functions in DNA repair and in replication of the lagging-strand chromosomal DNA (37). Its small N-terminal domain contains the 5′–3′ exonuclease activity. The large C-terminal Klenow fragment, which can be generated by partial digestion of the full-length polymerase (38,39), carries the polymerase and 3′–5′ exonuclease activities (40,41). Since MMEJ and SSA can generate large DNA deletions and both rely on DNA resection, we hypothesized that counteracting DNA resection with DNA pol I may favor the canonical NHEJ pathway over the MMEJ and SSA pathways, and thus decrease generation of large deletions. Thus, we tested targeting *E. coli* pol I to DSBs for counteracting DNA resection, to decrease the chances of generating large deletions (Figure 1A). In addition, doing so may increase filling in of possible 5′ overhangs generated by CRISPR/Cas9 and increase the proportion of TISs (Figure 1B), further refining the INDELs.

## MATERIALS AND METHODS

### Constructs

Constructs used in this study are described in Supplementary Table S1. The envelope plasmid pMD2.G for lentiviral pseudotyping was purchased from Addgene (Addgene 12259). Some plasmids used for this study will be available from Addgene (plasmid IDs 176234, 176235, 176236, 176237 and 176238). The remaining are available from the authors upon request. Sequences for primers used in this study are listed in Supplementary Table S2. sgRNA target sequences are listed in Supplementary Table S3.

### Cell culture

HEK293T (ATCC CRL-3216™) and HEK293T-derived *CLCN5* GFP reporter cells, described recently (42), were cultured in Dulbecco’s modified Eagle’s medium with 10% fetal bovine serum (FBS), 2 mM l-glutamine, 100 U/ml penicillin and 100 μg/ml streptomycin (Thermo Fisher Scientific) at 37°C in an incubator with 5% CO_2_. Human IMR90 cells (lung fibroblasts; ATCC CCL-186) were cultured in Eagle’s minimum essential medium supplemented with 10% FBS, 2 mM l-glutamine, 100 U/ml penicillin and 100 μg/ml streptomycin. Human CD34^+^ progenitor cells from mobilized peripheral blood (Lonza, catalog # 4Y-101C) were cultured in serum-free medium (Stem Cell Technology, catalog # 09605) supplemented with 1× StemSpan™ CD34^+^ Expansion Supplement (Stem Cell Technology, catalog # 02691). Human skeletal muscle myoblasts (Lonza, catalog # CC-2580) were cultured with an SkGM™-2 Skeletal Muscle Cell Growth Medium-2 BulletKit™ (Lonza, catalog # CC-3245).

### Transfection of HEK293T cells

HEK293T cells were transfected in 24-well plates using FuGENE HD (Promega, catalog # E2312). The day before transfection, 1.25 × 10^5^ cells were seeded in 24-well plates. For DNA transfection, 0.5 μg plasmid DNA was added to 50 μl of OPTI-MEM. In a different tube, 1.5 μl FuGENE HD was added to 50 μl OPTI-MEM. The two mixtures were mixed and incubated at room temperature for 15 min before adding to the cells whose medium was changed to OPTI-MEM just before DNA transfection. Twenty-four hours after transfection, the medium was changed to normal growth medium, and the cells were analyzed 72 h after transfection.

### Nucleofection of human primary cells

A Nucleofector™ 2b device (Lonza) was used for nucleofection of human primary cells. IMR90 cells, human myoblasts and human CD34^+^ hematopoietic cells were nucleofected with the Cell Line Nucleofector™ Kit R (Lonza, catalog # VCA-1001, program X-001), the Human Dermal Fibroblast Nucleofector™ Kit (Lonza, catalog # VPD-1001, program P-022) and the Human CD34^+^ Cell Nucleofector™ Kit (Lonza, catalog # VPA-1003, program U-008), respectively. Cell numbers for each nucleofection were 2 × 10^5^. We used 4.5 μg target plasmid DNA (expressing sgRNA/Cas9 or sgRNA/Cas9–Klenow) and 0.5 μg GFP-expressing plasmid DNA (CmiR0001-MR03, GeneCopoeia, Inc.) for each nucleofection, where the GFP-expressing plasmid DNA was used as an indicator for nucleofection efficiency. Transfected cells were checked for similar proportions of GFP-positive cells under a fluorescent microscope before further experiments.

### Knocking down *RBBP8* in human HEK293T cells and IMR90 cells

CRISPR/Cas9 nucleases were used to knock down *RBBP8* to investigate its functions in *CLCN5* mutation profiles induced by Cas9 or Cas9–Klenow. The sgRNA used was validated by Shou *et al.* (6) and is listed in Supplementary Table S3. The DNA ratio for *CLCN5* sgRNA- and *RBBP8* sgRNA-expressing plasmids was 1:2 to increase the chances of *RBBP8* knockdown. The DNA mixture was cotransfected into HEK293T cells by FuGENE HD and IMR90 cells by nucleofection as described earlier.

### Production of lentivirus-like particles

Lentivirus-like particles were produced as described previously (42). The packaging plasmid pspAX2-D64V-NC-COM has the aptamer-binding protein Com inserted in the nucleocapsid protein, and the sgRNA’s ST2 loop was replaced by a com aptamer to enable packaging of Cas9 ribonucleoprotein (RNP) into the lentiviral capsids via interactions between aptamer com and aptamer-binding protein Com. In brief, 5 million HEK293T cells were seeded in 10-cm tissue culture dishes. Twenty-four hours after cell seeding, the following DNA mixture was added to 500 μl of OPTI-MEM: 7.5 μg pspAX2-D64V-NC-COM, 7.5 μg plasmid DNA expressing *CLCN5* sgRNA and Cas9 (or Cas9–Klenow), and 3 μg pMD2.G. In parallel, 500 μl of OPTI-MEM was mixed with 54 μl of 1 mg/ml polyethylenimine (Polysciences, Inc.). The mixture was then incubated at room temperature for 15 min before being added to the cells. After 24 h of transfection, fresh OPTI-MEM was added to the cells, and the medium containing virus-like particles was collected 48 h later. A p24-based ELISA was used to determine particle concentrations (Cell Biolabs, QuickTiter™ Lentivirus Titer Kit, catalog # VPK-107).

### Transduction of lentivirus-like particles

The virus-like particles were added to the cells in the presence of 8 μg/ml polybrene to transduce the lentivirus-like particles into HEK293T cells or *CLCN5* GFP reporter cells. Particles up to 200 ng p24 of particles were added to 2.5 × 10^4^ cells grown in 24-well plates. Twenty-four hours after transduction, the medium was changed to normal growth medium. GFP expression in *CLCN5* GFP reporter cells could be detected 36 h after transduction. The cells were collected for DNA isolation 72 h after transduction.

### PCR amplification of target DNA for INDEL profile analyses

Genomic DNA was isolated using the DNeasy Blood & Tissue Kit (Qiagen, Germantown, MD) according to the manufacturer’s instructions. The primer sequences used for amplifying target DNAs are listed in Supplementary Table S2. The genomic DNA template input for PCR was up to 0.5 μg. For samples with low DNA concentrations, 0.2 μg DNA was used. To reduce amplification bias, predetermined minimal cycle numbers (25–30) were used. The proofreading CloneAmp HiFi PCR Premix (Takara, Mountain View, USA; catalog # 639298) was used for PCR.

### Off-target analysis

Four potential off-targets for hemoglobin subunit beta (*HBB*) sgRNA1 were analyzed to compare off-target activities between Cas9 and Cas9–Klenow. These include G1-OT4 and G1-OT5, as reported by others (43), and HBD and Off-8, as we reported previously (44). The regions of the predicted off-targets were amplified with their respective specific primers (Supplementary Table S2) and subjected to next-generation sequencing (NGS) analyses.

### NGS and data analyses

NGS was done by Genewiz, Inc. (Morrisville, NC) using their ‘Amplicon EZ’ service. Approximately 50 000 reads were obtained per sample. After removing the 3′ linker and 5′ barcode sequences, the resulting reads were uploaded to the online software programs Cas-Analyzer (45) and CRISPResso2 (46) for mutation analyses. The two programs gave similar results in most cases, except that CRISPResso2 did not perform well when the INDEL rates were <5%. Data presented here were analyzed with Cas-Analyzer unless otherwise stated.

### Single-molecule real-time sequencing (PacBio)

Single-molecule real-time (SMRT) sequencing was performed to detect large deletions targeting the human *CLCN5* gene. A region of 4862 bp was amplified by LongAmp^®^ Hot Start Taq 2× Master Mix (New England Biolabs, catalog # M0533) with primers hCLCN5-F3 and hCLCN5-R3. DNA was submitted to the GCB Sequencing and Genomic Technologies Shared Resource (Duke University, Durham, NC) for SMRT sequencing (Sequel I). One SMRTcell was used for eight barcoded samples. Demultiplexed circular consensus (CCS) reads received from the sequencing center were used to search for large deletions by comparing with the reference sequence through R programming. Specifically, all sequences were searched for the presence of a 25-bp 5′ sequence and a 25-bp 3′ sequence (see Supplementary Table S2 for sequences), each allowing a 2-nt difference (92% identity) to accommodate possible sequencing errors. These two regions are at the 5′ and 3′ ends of the sequenced DNA. The distances between the two 25-bp regions were then calculated for each read. Reads with distances that differed from the reference sequence and without intact sgRNA target sites were regarded as reads with deletions. The counts and the sequences of the reads with deletions were listed for further analysis.

### Statistical analysis

GraphPad Prism software (V5) was used to perform *t*-tests, ANOVA and chi-square tests. *P* < 0.05 was regarded as statistically significant. Means and standard errors of the mean are reported.

## RESULTS

### Targeting *E. coli* DNA pol I to DNA DSBs increased 1-bp deletions and TISs, and decreased large deletions induced by CRISPR/Cas9

We targeted *E. coli* DNA pol I to DNA DSBs by fusing it to the C-terminus of SpCas9, with a linker peptide used in EvolvR in *E. coli* (47) and yeast (48). To improve fusion protein expression in human cells, we modified the codons of the polA gene that codes for pol I (49). We used the fusion protein to target the 5′ untranslated region (UTR) of human chloride voltage-gated channel 5 (*CLCN5*) in GFP reporter cells that we developed for sensitively detecting genome editing activities (42). When *CLCN5* is mutated, it causes Dent’s disease, a rare kidney disorder (50). Our GFP reporter cells do not express GFP because the GFP reading frame is disrupted by inserting the *CLCN5* sgRNA target sequence (from the *CLCN5* 5′ UTR region) between the start codon and the second codon of the GFP coding sequence. GFP will only be expressed if genome editing restores the GFP reading frame via in-frame INDELs (∼1 in 3 chance). We observed similar GFP-positive cells in cells treated with *CLCN5* gRNA/Cas9 and *CLCN5* gRNA/Cas9–pol. Targeted deep sequencing confirmed genome editing at the target site by *CLCN5* gRNA/Cas9–pol (Supplementary Figure S1), demonstrating the functionality of the Cas9–pol fusion protein.

We transfected plasmid DNA expressing *CLCN5* gRNA/Cas9 or *CLCN5* gRNA/Cas9–pol (targeting human *CLCN5* 5′ UTR) into HEK293T cells and observed similar INDEL rates generated by Cas9 and Cas9–pol (11.6 ± 1.6%, *N* = 3 for Cas9; 14.5 ± 0.7%, *N* = 3 for Cas9–pol, *P* = 0.16). This suggests that the fusion did not impair Cas9 activity.

We then analyzed the INDEL profiles induced by Cas9 and Cas9–pol targeting *CLCN5*. Cas9–pol caused a significant increase in 1-bp deletions, a decrease in >1-bp deletions (Figure 1C) and an increased ratio of 1-bp TIS versus 1-bp non-TIS (Figure 1D). The data support our hypothesis that targeting pol I to DSBs favors the generation of short deletions over long deletions and TIS over non-TIS.

### Targeting polymerase to DSBs is necessary for modulating TIS but not deletion size

**Figure 2.**
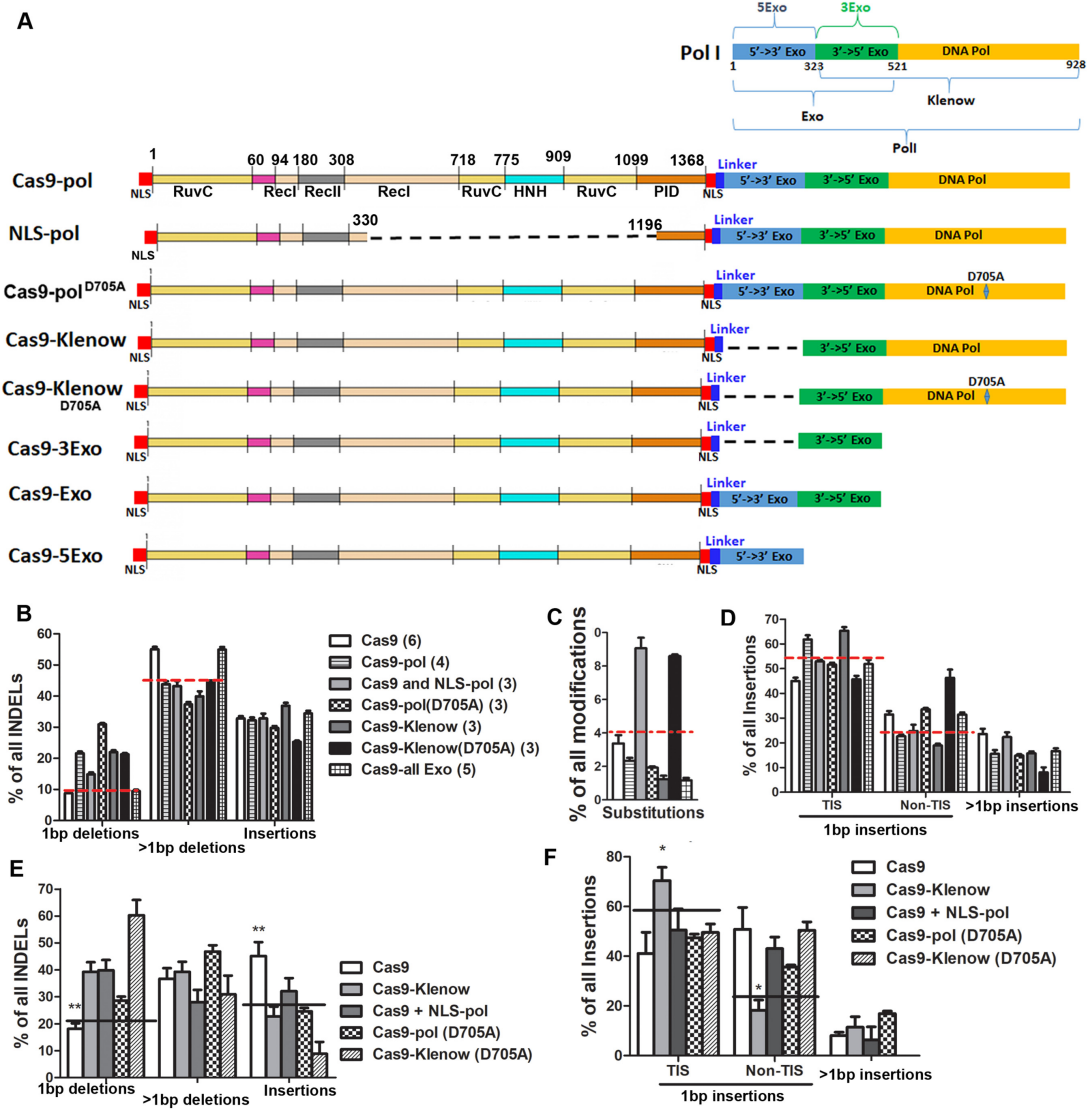
Effects of pol I mutation on Cas9-induced DNA mutation profile. (**A**) Cas9–pol and various mutant fusion proteins tested. Dashed lines indicate the deleted regions. (**B**) Effects of different DNA pol I mutants on deletion profiles targeting the *CLCN5* gene in HEK293T cells. (**C**) Analysis of substitution rates of various fusion proteins (analyzed with CRISPResso2). (**D**) Effects of different DNA pol I mutants on insertions targeting the *CLCN5* gene in HEK293T cells. (**E**) Effects of different DNA pol I mutants on deletion profiles targeting the *CLCN5* gene in IMR90 cells. (**F**) Effects of different DNA pol I mutants on insertions targeting the *CLCN5* gene in IMR90 cells. In panels (B)–(D), the same sample labels are used and numbers in parentheses indicate biological replicates. Groups above the dashed lines showed statistical significance compared to those below the lines (Bonferroni post-tests following two-way ANOVA). In panels (E) and (F), experiments were replicated three times for all groups. * and ** indicate *P* < 0.05 and *P* < 0.01, respectively, between the indicated group and all other groups (Bonferroni post-tests following two-way ANOVA).

To test whether targeting the polymerase to DSBs is necessary for the observed effects, we made NLS–pol protein, a deletion mutant of Cas9–pol that contained full-length pol I and the nuclear localization signals (NLS), but lacked most of the Cas9 functional domains (part of REC and RuvC, the whole HNH and part of the PAM-interacting domain of Cas9) (Figure 2A). Since NLS–pol contained the NLS and linker sequences of Cas9–pol, it was expected to fold properly and be targeted to the nuclei. However, it should not have Cas9’s DNA binding and nuclease activities due to the deletion of multiple Cas9 domains, as observed previously (51). We co-expressed Cas9, *CLCN5* gRNA and NLS–pol in HEK293T cells. Cells treated with Cas9 and NLS–pol had 1-bp deletion percentages between those of Cas9-treated cells and Cas9–pol-treated cells (Figure 2B), and had increased DNA substitution rates in a region 20 bp 5′ and 20 bp 3′ of the predicted cleavage site for unknown mechanisms (Figure 2C). Importantly, co-expression of Cas9 and NLS–pol did not increase TIS to the level of Cas9–pol (Figure 2D).

To ask whether the polymerase activity is necessary for the observed effects, we fused Cas9 to various mutants and truncated pol proteins, including pol^D705A^ with the polymerase activity inactivated (52,53), the Klenow fragment, Klenow^D705A^ with the polymerase activity inactivated, the 5′ exonuclease domain (5Exo), the 3′ exonuclease domain (3Exo), and both of the 5′ exonuclease and the 3′ exonuclease domains (Exo) (Figure 2A). We targeted *CLCN5* in HEK293T cells using these Cas9 fusion proteins. All fusion proteins without the DNA polymerase domain failed to increase 1-bp deletions and decrease >1-bp deletions. However, all fusion proteins with the DNA polymerase domain—regardless of whether the polymerase activity was inactivated—increased 1-bp deletions and decreased >1-bp deletions (Figure 2B). Since Cas9-5Exo, Cas9-3Exo and Cas9-Exo all showed very similar mutation profiles (Supplementary Figure S2), they were treated as one group (Cas9-all Exo) in Figure 2B. Thus, the polymerase domain, rather than the polymerase activity, was enough to perturb the ratio of 1-bp and >1-bp deletions.

Only pol I and the Klenow fragment increased 1-bp TIS and decreased 1-bp non-TIS (Figure 2D), consistent with the need of polymerase-mediated filling in for generating TIS. The same results were seen when 2- and 3-bp TISs were analyzed (Supplementary Figure S3). Compared with pol I, the Klenow fragment showed stronger effects on increasing 2- and 3-bp TISs. We reason that this was most likely because the Klenow fragment lacks the 5′ > 3′ exonuclease domain present in pol I, and the 5′ > 3′ exonuclease domain may remove the 5′ overhangs and favor the generation of deletions.

We similarly targeted *CLCN5* in human primary IMR90 cells with various fusion proteins. In these cells, all fusion proteins with the Klenow domain (with or without polymerase activity) significantly increased 1-bp deletion frequency but decreased insertion frequency rather than >1-bp deletion frequency (Figure 2E). Again, targeting the polymerase activity to the DSBs increased TIS (Figure 2F).

We examined the relationship between the mutation profiles and the overall INDEL rates targeting the *CLCN5* locus by Cas9. Overall INDEL rates did not affect mutation profiles (Supplementary Figure S4). Thus, the observed effects of fusing pol I or the Klenow fragment to Cas9 on DNA mutation profiles could not be explained by possible effects on Cas9 cleavage activity. The most likely mechanism is via interfering with local cellular DNA repair machineries.

Altogether, targeting polymerase to DSBs promoted the generation of TIS and 1-bp deletions. Fusing polymerase to Cas9 is beneficial, since doing so increases local polymerase concentrations and decreases possible interference with other endogenous DSBs—especially since in many experimental settings, controlled amounts of genome editing effectors rather than overexpressed ones are used for genome editing.

### DNA resection was involved in the perturbation of 1-bp versus >1-bp deletions

The observations that pol^D705A^ and Klenow^D705A^ also favored the generation of 1-bp deletions over >1-bp deletions prompted us to examine whether DNA resection was involved in these effects. We thus examined the mutation profiles targeting the *CLCN5* locus in cells with and without *RBBP8* (expressing CtIP protein) knockdown. Considering the possibility of only disrupting the expression from one allele, we use the term knockdown rather than knockout. We used the previously validated *RBBP8* sgRNA (6) to mediate the mutation of this gene. *RBBP8* sgRNA-expressing DNA was cotransfected with *CLCN5* sgRNA-expressing DNA into HEK293T cells. The sgRNA-expressing constructs also contained Cas9- or Cas9–Klenow-expressing cassettes (see Supplementary Table 1 for DNA constructs used). INDEL rates of the cotransfected genes (*RBBP8* and *CLCN5*) were very similar (Supplementary Table S4), consistent with cotransfection. We observed quite different overall INDEL rates in Cas9- and Cas9–Klenow-treated cells, for unknown reasons. However, since DNA mutation profiles were independent of overall INDEL rates (Supplementary Figure S4), this INDEL rate difference is unlikely to affect our mutation profile analyses.

**Figure 3.**
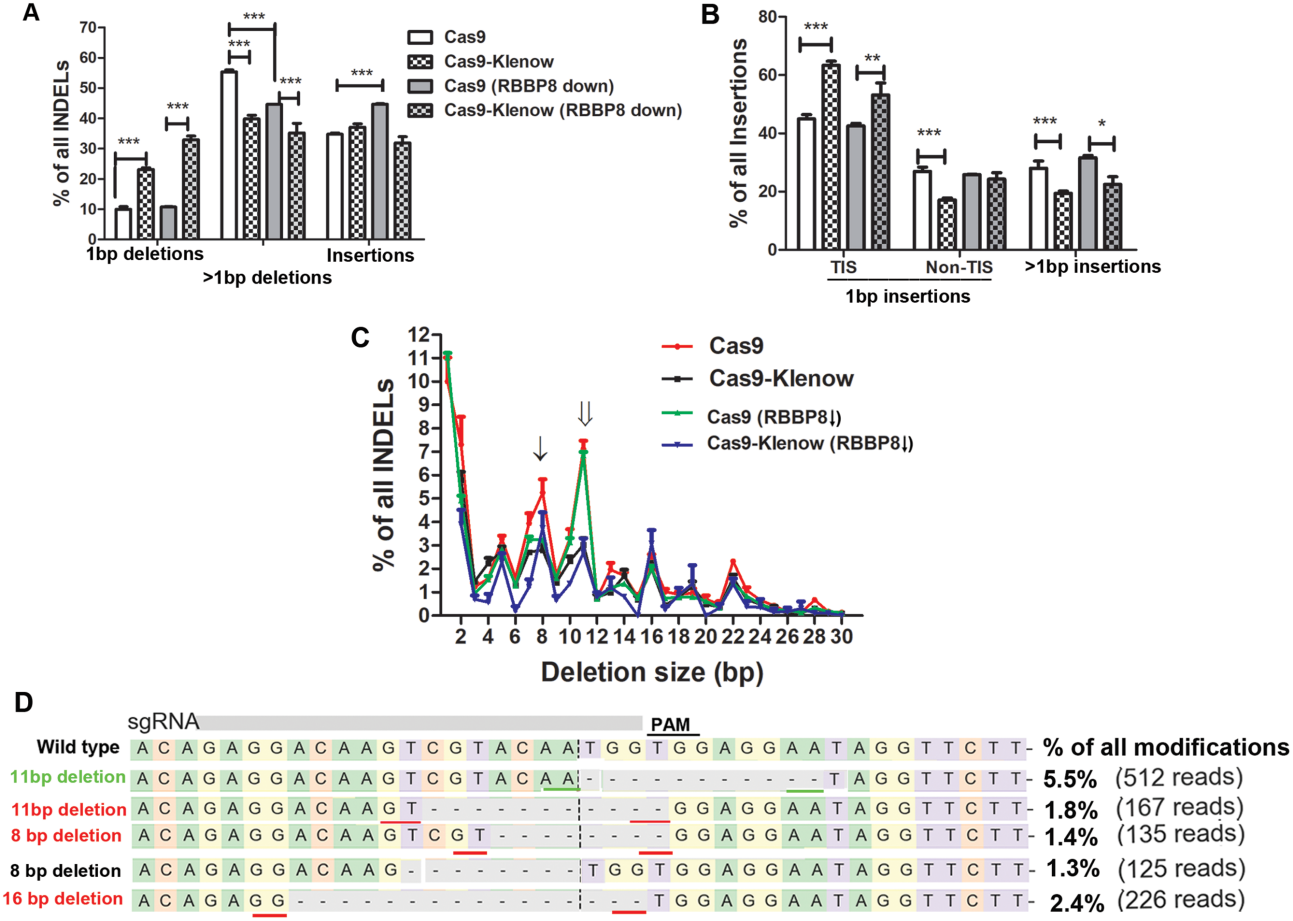
Effects of DNA resection inhibition on Cas9-induced DNA mutation profile. (**A**) Effects of *RBBP8* knockdown on Cas9-induced DNA mutation profiles targeting *CLCN5* in HEK293T cells. (**B**) Effects of *RBBP8* knockdown on Cas9-induced insertions targeting *CLCN5* in HEK293T cells. For panels (A) and (B), replicate numbers were 6 for Cas9 and 3 for the remaining groups. *, ** and *** indicate *P* < 0.05, *P* < 0.01 and *P* < 0.001, respectively, between the indicated groups (Bonferroni post-tests following two-way ANOVA). (**C**) Effects of *RBBP8* knockdown on deletions with various sizes targeting *CLCN5* in HEK293T cells. The single and double arrows indicate the resection-dependent and -independent deletions, respectively. (**D**) Most frequently observed deletions generated by Cas9 targeting *CLCN5* in HEK293T cells. Dashed lines indicate deletions. Microhomology is underlined. In the online version, green lines indicate microhomology at the predicted cleavage size, which is indicated by a vertical dashed line.

Knocking down *RBBP8* did not affect the percentage of 1-bp deletions, but significantly decreased the percentage of >1-bp deletions and increased the percentage of insertions when targeting *CLCN5* in HEK293T cells (Figure 3A). These results are consistent with the suppression of DNA resection, which may cause increased insertions. These observations are similar to those from the Repair-seq, published while our paper was under revision (54). Importantly, in cells with *RBBP8* knocked down, the Klenow fragment decreased >1-bp deletions to a smaller extent than in cells without *RBBP8* knockdown, suggesting that *RBBP8* is involved in Klenow-mediated decrease of >1-bp deletions. TIS of 1-bp was not significantly changed in *RBBP8* knockdown HEK293T cells, and Klenow fragment increased 1-bp TIS (Figure 3B).

We also examined the *CLCN5* mutation profiles in IMR90 cells with and without *RBBP8* knockdown. Knocking down *RBBP8* in IMR90 cells increased Cas9-induced 1-bp deletions, an effect similar to fusing the Klenow fragment to Cas9 in normal IMR90 cells (Supplementary Figure S5). Doing so also increased 1-bp TIS and decreased 1-bp non-TIS. In *RBBP8* knockdown cells, the Klenow fragment had no effects on 1-bp deletions, >1-bp deletions or TIS. These observations suggest that knocking down *RBBP8* and fusing the Klenow fragment to Cas9 may interfere with similar pathways in IMR90 cells. Altogether, these data suggest that interfering with DNA resection is at least one mechanism for pol I or the Klenow fragment’s effects on Cas9 DNA mutation profiles.

For deletions from 1 to 30 bp targeting *CLCN5* in HEK293T cells, except for 1-bp deletions, 11- and 8-bp deletions were the most frequently observed (Figure 3C). The frequencies of the 11-bp deletions were unaffected by *RBBP8* knockdown (Figure 3C), suggesting that other mechanisms may also be involved in the generation of these deletions. These deletions were greatly suppressed by fusing the Klenow fragment to Cas9. The frequency of 8-bp deletions was decreased by *RBBP8* knockdown, and not further decreased by the Klenow fragment. This observation further supports the concept that one way in which the Klenow fragment affects the Cas9 DNA mutation profile is via interfering with DNA resection.

Microhomology was noted around the deletion when examining the most frequently deleted sequences in Cas9-treated cells (Figure 3D, underlined). The most frequently observed 11-bp deletion had microhomology (underlined green in the online version) at the very end of the predicted cleavage site (dashed line). In this case, DNA synthesis could be initiated without the need for 3′ flap endonucleases such as XPF–ERCC1 (55,56) to remove the unmatched 3′ flap during MMEJ. This possibly explains why the 11-bp deletion was most frequently seen in Cas9-treated cells. *RBBP8* knockdown had no effects on the frequency of this deletion, and there could be other unknown proteins to generate similar deletions under resection inhibition. In general, the common deletions either had microhomology around the deletion or had one end at the predicted cleavage site, or both.

### Cas9–Klenow increased 1-bp deletions on multiple loci in multiple human cell types

We then examined whether effects of pol I or the Klenow fragment on DNA mutation profiles were target sequence or cell type specific. We used the Cas9–Klenow fusion protein in subsequent experiments, given its smaller size and prominent effects on increasing 1-bp deletions and TISs. We examined four more loci, including Duchenne muscular dystrophy (*DMD*) exon 53 and *DMD* exon 44 (537 kb away from each other), the 5′ coding region of *HBB* and an intergenic locus intragenic 1 (GRCh38.p13, chromosome 20, 32752960–32752979). *DMD* exons 53 and 44 were picked because targeting these exons with a single-cut sgRNA might restore dystrophin in patients with DMD caused by exon deletion (57,58). The *HBB* 5′ coding region was picked for possible application of genome editing in treating sickle cell disease. In addition, we previously targeted this region with CRISPR/Cas9 to examine Cas9-induced gene conversion in human somatic cells (44). The intragenic 1 locus was picked to rule out possible contributions of target gene products on the observed effects. In addition to HEK293T cells, we targeted various loci in human primary fibroblast IMR90 cells, human CD34^+^ hematopoietic stem cells and human primary myoblasts for a total of eight loci/cells (Table 1).

**Table 1.** Effects of Cas9–Klenow on deletions, total insertions and 1-bp TIS

Locus/cell	1-bp deletions (% of all INDELs)		>1-bp deletions (% of all INDELs)		Insertions (% of all INDELs)		1-bp TIS (% of all insertions)	
	Cas9	Cas9–Klenow	Cas9	Cas9–Klenow	Cas9	Cas9–Klenow	Cas9	Cas9–Klenow
*CLCN5*/293T	**9.9 ± 0.92**	23.1 ± 0.53***	*55.3 ± 0.66*	39.8 ± 1.2***	**34.8 ± 0.45**	37.1 ± 1.1*	**45.0 ± 1.39**	65.4 ± 1.44***
*CLCN5*/IMR90	**18.1 ± 2.00**	39.3 ± 3.59**	36.7 ± 3.90	39.3 ± 3.72	*45.1 ± 5.16*	22.7 ± 3.60*	**41.2 ± 8.57**	70.4 ± 5.33*
*DMD53*/293T	**8.4 ± 0.14**	19.8 ± 0.45***	*64.4 ± 1.3*	51.6 ± 0.37***	27.2 ± 1.40	28.5 ± 0.31	**34.0 ± 4.17**	52.6 ± 0.68*
*DMD44*/myoblasts	**11.2 ± 1.95**	28.8 ± 2.71**	*75.8 ± 2.06*	57.4 ± 2.65**	13.0 ± 1.41	13.7 ± 0.67	**32.6 ± 9.27**	66.46 ± 6.63*
*HBB*/hematopoietic cells	**37.4 ± 4.11**	59.6 ± 3.05*	*56.7 ± 4.59*	36.4 ± 2.90*	4.2 ± 1.10	4.0 ± 0.42	**21.2 ± 6.02**	46.7 ± 5.90*
*HBB*/IMR90	**40.8 ± 9.99**	81.2 ± 5.47*	*55.9 ± 10.64*	14.7 ± 6.32*	2.3 ± 1.02	4.5 ± 2.34	Too few	Too few
*HBB*/293T	**13.3 ± 0.84**	46.7 ± 1.34***	*65.1 ± 0.59*	41.1 ± 1.29***	*19.6 ± 1.03*	10.3 ± 0.16**	41.9 ± 1.14	39.1 ± 3.33
Intragenic 1/IMR90	**14.3 ± 2.1**	33.7 ± 1.22**	47.4 ± 2.1	48.0 ± 1.41	*38.3 ± 0.60*	18.2 ± 0.47***	**23.3 ± 1.93**	50.3 ± 0.96**

*, ** and *** indicate *P* < 0.05, 0.01 and 0.0001, respectively, in two-tailed *t*-tests. Bold, italic and roman values indicate increased, decreased and unchanged results in the Cas9–Klenow group, respectively. Since Klenow fusion changed the total insertion percentages significantly in five loci/cells, 1-bp TIS was expressed as % of all insertions.

In all cases, targeting the Klenow fragment to DSBs significantly increased 1-bp deletions (Table 1), from an average of 19.2 ± 4.48% to 41.5 ± 7.28% in the eight analyzed loci/cells. In *HBB*/IMR90, 81.2 ± 5.47% of all INDELs generated by Cas9–Klenow were 1-bp deletions. The increase of 1-bp deletions was accompanied by four possible phenomena: (i) decreased >1-bp deletions (*DMD53*/293T, *DMD44*/myoblasts, *HBB*/hematopoietic cells and *HBB*/IMR90); (ii) decreased insertions (*CLCN5*/IMR90, intragenic 1/IMR90); (iii) decreased >1-bp deletions and insertions (*HBB*/293T); and (iv) decreased >1-bp deletions and increased total insertions (*CLCN5*/293T). Although effects of the Klenow fragment on insertions varied, it caused significantly decreased >1-bp deletion in six of eight loci/cells. Targeting the *HBB* locus in IMR90, HEK293T and hematopoietic cells showed different possible mechanisms for the increased 1-bp deletions.

**Figure 4.**
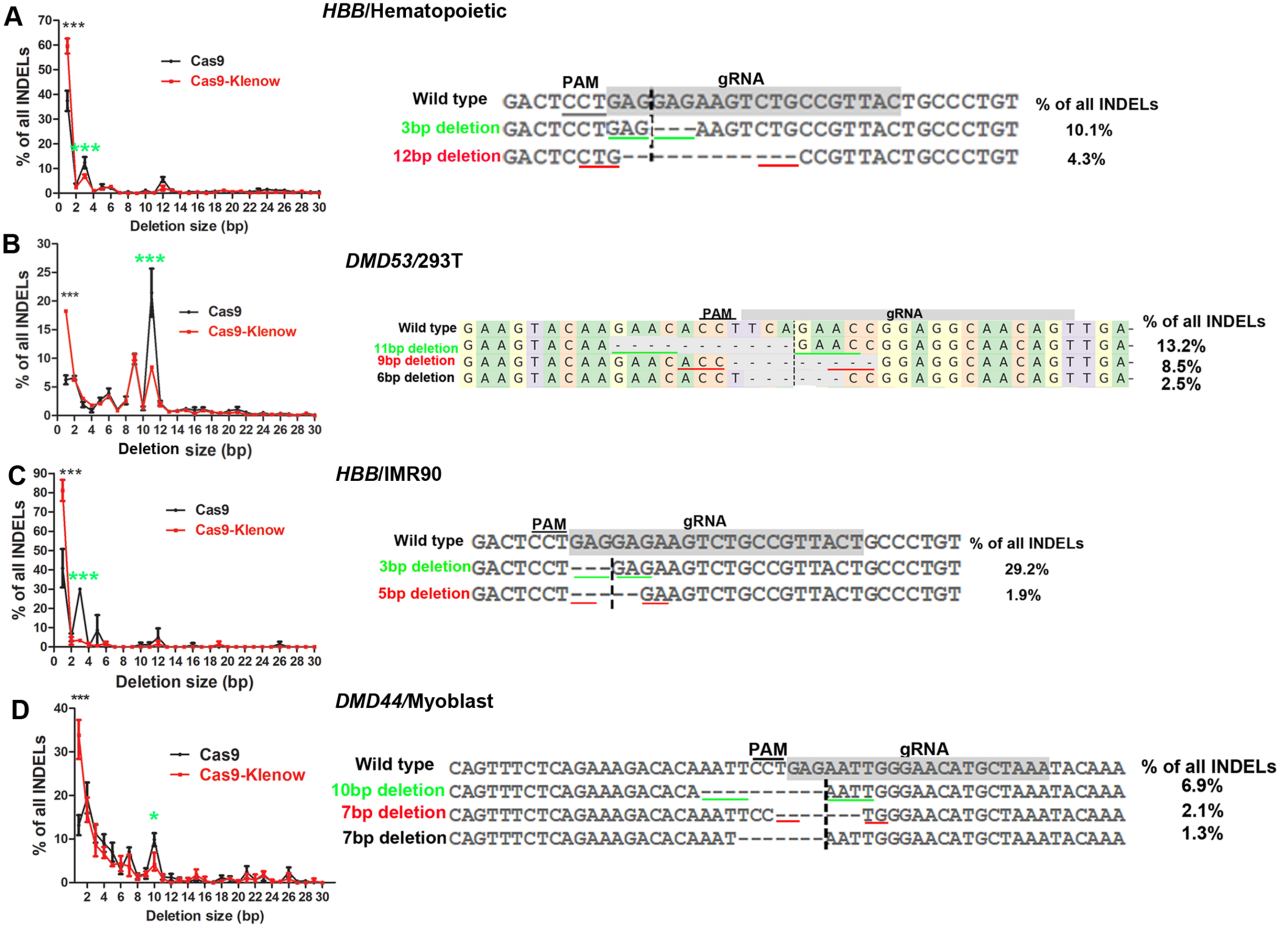
Examination of suppressed deletions targeting different genes in different cells. (**A**) Targeting the *HBB* gene in hematopoietic cells. (**B**) Targeting the *DMD* exon 53 in HEK293T cells. (**C**) Targeting the *HBB* gene in IMR90 cells. (**D**) Targeting the *DMD* exon 44 in human myoblasts. * and *** indicate *P* < 0.05 and *P* < 0.001, respectively, between Cas9 and Cas9–Klenow (Bonferroni post-tests following two-way ANOVA). The left images show the peaks of deletions and the right images show the most frequently observed deletions. Microhomology is underlined. Green lines (online version) indicate microhomology at the predicted cleavage size, which is indicated by a vertical dashed line. Red lines (online version) indicate microhomology away from the predicted cleavage site. Each group has three biological replicates.

In all loci/cells except for *CLCN5*/IMR90, which had no evident deletion peaks (Supplementary Figure S6), Cas9–Klenow most significantly decreased the percentages of >1-bp deletions with microhomology at the predicted cleavage site (underlined by green lines in the online version). These were usually the highest >1-bp deletion peaks generated by Cas9 (Figures 3D and Figure 4, and Supplementary Figure S6). The smaller >1-bp deletion peaks either had no microhomology but were located at the predicted cleavage site (dashed vertical line) or had microhomology away from the cleavage site (underlined by red lines in the online version). These observations suggest that MMEJ events that do not require removal of 3′ flaps were most sensitive to suppression by the Klenow fragment.

### Cas9–Klenow increased TIS on multiple loci in multiple human cell types

We then examined the effects of the Klenow fragment on TIS in these loci/cells. We focused on 1-bp TIS since 2- and 3-bp TISs were rare in the ∼50 000 reads we analyzed in most loci/cells. Since in several cases the Klenow fragment greatly changed the percentage of overall insertions, we compared the percentages of TIS in all insertions rather than all INDELs. Except for one locus/cell (*HBB*/IMR90), which had too few insertions and therefore was not analyzed, six of seven loci/cells showed a significant increase in the proportion of TIS in all insertions (Table 1). Only *HBB*/293T locus/cell showed similar TIS percentages between Cas9 and Cas9–Klenow, and ∼80% of 1-bp insertions were TIS. This observation suggests that in the case of *HBB*/293T locus/cell, the endogenous polymerases were very efficient in filling in the 5′ overhangs, explaining why Cas9–Klenow did not further increase the proportion of TIS.

Fusing the Klenow fragment to Cas9 caused a significant reduction of total insertions in three loci/cells (*CLCN5*/IMR90, *HBB*/293T and intragenic 1/IMR90). In two of these (*CLCN5*/IMR90 and intragenic 1/IMR90), non-TIS insertions contributed to 100% of the decreased insertions.

### Fusing Klenow to Cas9 decreased CRISPR/Cas9-induced large DNA deletions

**Figure 5.**
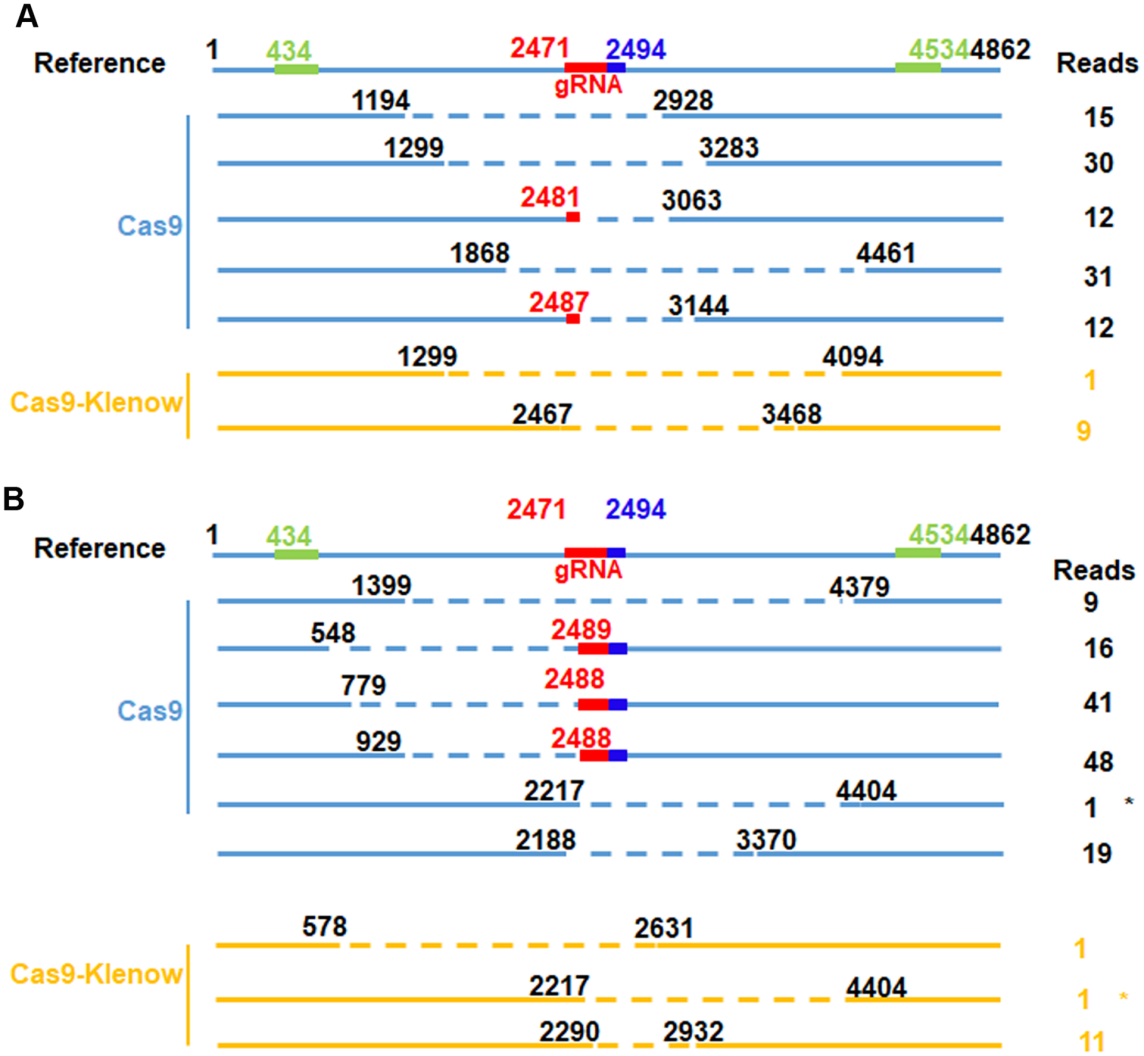
Comparison of large deletions generated by Cas9 and Cas9–Klenow targeting *CLCN5* gene in human cells. (**A**) Large deletions in HEK293T cells. (**B**) Large deletions in IMR90 cells. The data were combined from three replicates for each cell type. In the online version, the green regions indicate the two 25-bp sequences used for calculating distance for deletion detection; the red region indicates the sgRNA target and the blue region indicates the PAM. The asterisks indicate the identical deletions independently observed in two different treatments. See Table 2 for results of chi-square tests of the data.

We next analyzed large deletions targeting *CLCN5* in HEK293T cells. We designed a pair of primers amplifying a region of 4862 bp, with the sgRNA target sequence in the middle of the amplicon (Figure 5). We then generated lentivirus-like particles containing the Cas9 RNPs or the Cas9–Klenow RNPs as we recently reported (42). Similar percentages of GFP-positive cells resulted after treating the *CLCN5* GFP reporter cells with the two types of RNPs, suggesting equivalent genome editing activities.

We then treated HEK293T cells with Cas9 RNPs or Cas9–Klenow RNPs. We amplified the DNA 72 h after treatment and performed SMRT sequencing (PacBio). We found more reads with >0.5-, >1- and >2-kb deletions in Cas9-treated cells than in Cas9–Klenow-treated cells (Table 2 and Figure 5).

**Table 2.** Large deletions generated by Cas9 and Cas9–Klenow targeting *CLCN5*

	HEK293T cells			IMR90 cells		
	Cas9	Cas9–Klenow	No sgRNA	Cas9	Cas9–Klenow	No sgRNA
**Total CCS** ^a^	23 477	11 497	12 040	19 147	29 793	11 417
**CCS with >0.5-kb deletions**	100	10	0	151	13	1
**CCS with >1-kb deletions**	76	1	0	132	2	1
**CCS with >2-kb deletions**	28	1	0	50	2	0
**Chi-square test**	*P* < 0.0001		*P* < 0.0001			

^a^Only those CCSs containing both the 5′ and 3′ index sequences (green boxes in online version of Figure 5) were analyzed. CCS: circular consensus.

We further targeted *CLCN5* in IMR90 cells by nucleofection of plasmid DNA expressing *CLCN5* sgRNA/Cas9 or *CLCN5* sgRNA/Cas9–Klenow. Although Cas9-treated cells had lower short INDEL rates than Cas9–Klenow-treated cells (see Table 3, which used the same DNA samples for NGS analysis), they had more >0.5-, >1- and >2-kb deletions than Cas9–Klenow-treated cells.

**Table 3.** INDEL rates (%) of Cas9 and Cas9–Klenow in human primary cells

Locus/cell	Cas9	Cas9–Klenow	Background
*HBB/*IMR90	2.6 ± 0.50	6.2 ± 0.87*	0.02
*HBB/*CD34^+^ cells	0.77 ± 0.03	1.6 ± 0.09***	0.01
*CLCN5/*IMR90	4.9 ± 0.50	7.8 ± 0.79*	0.09
*DMD44*/myoblasts	11.9 ± 1.8	12.8 ± 0.4	0
Intragenic 1/IMR90	0.77 ± 0.12	5.8 ± 0.46***	N/A

* and *** indicate *P* < 0.05 and *P* < 0.0001, respectively, in two-tailed *t*-tests (*n* = 3).

Among the CCS reads with >0.5-kb deletions, the deleted regions either spanned or involved the sgRNA target site (Figure 5), confirming that they were on-target deletions. We only observed limited types of deletions. Cas9-treated cells generated more types of deletions and often more CCS reads for each type of deletion than Cas9–Klenow-treated cells. Both phenomena could be explained by Cas9 being more prone to generate large deletions than Cas9–Klenow. Multiple CCS reads for the same type of deletion could be the result of a single deletion event in one cell or multiple deletion events in multiple cells. Our observations of the same deletion type in Cas9-treated and Cas9–Klenow-treated IMR90 cells (indicated by * in Figure 5B) supported the possibility of multiple deletion events for the same type of deletion. Alternatively, Cas9 treatment could have induced the large deletions sooner after treatment than Cas9–Klenow treatment, which increased the representation of the deletions.

### Cas9–Klenow increased INDEL rates in human primary cells

Whereas Cas9 and Cas9–Klenow (or Cas9–pol) generated similar levels of INDEL rates in HEK293T cells, in human primary cells Cas9–Klenow treatment generated significantly higher INDEL rates in five of six loci/cells (Table 3). In general, the INDEL rates were relatively low in primary cells, for unknown reasons. We confirmed that the observed INDELs were authentic INDELs, since the background INDELs of cells treated with nontargeting sgRNA were very low, and all the INDELs were around the predicted cleavage sites. We included 1/10 GFP-expressing plasmid DNA in the nucleofection experiments and observed >50% GFP-positive cells. Thus, the low INDEL rates were not due to low nucleofection efficiency. We did observe noteworthy cell death during the 72 h of culture time after nucleofection. We postulate that many Cas9- or Cas9–Klenow-positive cells might have been lost due to the toxic effects of constantly generating DSBs, as in human embryonic stem cells (59,60).

### Cas9–Klenow did not increase DNA substitution rates or off-targets

Next, we tested whether targeting DNA polymerase to DSBs could increase DNA mutation rates around DSBs. We analyzed DNA substitution rates in a 40-bp region around the predicted cleavage sites (20 bp on each side) because (i) MRE11 nicks 15–20 nt away from the DSBs (18), (ii) pol I showed a processivity of 15–20 nt (61) and (iii) EvolvR (the fusion protein between Cas9 nickase and error-prone DNA pol I) showed a mutation window of 15–20 bp (47). Cas9–Klenow treatment did not cause increased DNA substitution rates (Table 4). In addition, similar DNA substitution rates were also observed in negative controls with nontargeting sgRNAs, which suggests that the observed DNA substitutions were mainly from cell heterogeneity, or PCR and sequencing errors. We concluded that targeting DNA polymerase to DSBs did not increase DNA substitution rates.

**Table 4.** Comparison of DNA substitution percentages caused by Cas9 and Cas9–Klenow treatments^a^

Locus/cell	Cas9	Cas9–Klenow	*P*-value	Cas9 nontargeting sgRNA
*CLCN5*/293T	3.38 ± 0.49 (6)	1.23 ± 0.18 (3) ↓	0.021205*	N/D
*DMD53*/293T	2.44 ± 0.69 (3)	2.14 ± 0.624 (3)	0.245357	N/D
Intragenic 1/IMR90	2.52 ± 0.12 (3)	2.724 ± 0.09 (3)	0.676036	N/D
*HBB*/CD34	9.02 ± 0.11 (3)	9.18 ± 0.09 (3)	0.400207	9.25 (1)
*HBB/*293T	1.97 ± 0.05 (3)	1.86 ± 0.09 (3)	0.341773	N/D
*HBB*/IMR90	0.95 ± 0.02 (3)	0.98 ± 0.0498 (3)	0.760798	0.99 (1)
*DMD44*/myoblasts	1.77 ± 0.14 (4)	1.94 ± 0.07 (3)	0.929889	1.85 (1)
*CLCN5*/IMR90	15.69 ± 0.27 (3)	16.01 ± 0.26 (3)	0.43238	16.84 (1)

^a^DNA substitution rates were calculated as percentages of reads with substitutions over total reads. Numbers of replicated experiments are listed in parentheses. * indicates statistically significant (p<0.05).

We further tested the effects of fusing the Klenow fragment to Cas9 on possible off-targets of Cas9. We targeted the *HBB* 5′ coding region in human IMR90 cells and detected INDEL rates at four potential off-targets predicted based on sequence similarity (Supplementary Figure S7). We did not observe off-targets in Cas9- and Cas9–Klenow-treated cells using targeted NGS (<0.5%); this is the detection limit of NGS, currently one of the most sensitive off-target detection methods. Thus, fusing the Klenow fragment to Cas9 did not increase off-targets to a detectable level by NGS. Since the Klenow fragment did not cause detectable off-targets on sequences similar to the authentic target, it is less likely to cause random off-targets on sequences that are not similar to the authentic target.

We then examined Klenow’s effects on off-targets in a third way. We previously developed HEK293T-derived GFP reporter cells for detecting CRISPR/Cas9-induced INDELs in an *HBB* sickle mutant sequence (62), which differs by 1 nt from the *HBB* sgRNA we used in this study and is an ‘off-target’ for the *HBB* sgRNA. These cells contain *HBB* sgRNA authentic targets in the endogenous *HBB* gene and off-targets in the integrated GFP reporter cassette. We targeted the endogenous *HBB* gene with the perfectly matching *HBB* sgRNA and examined INDEL rates in the sickle mutant sequence as an off-target. Cas9- and Cas9–Klenow-treated cells had similar INDEL rates on the endogenous *HBB* target (21.47 ± 0.80%, *N* = 3 for Cas9; 21.70 ± 1.10%, *N* = 3 for Cas9–Klenow, *P* = 0.8723) and the integrated sickle mutant sequence (10.80 ± 0.80%, *N* = 3 for Cas9; 12.73 ± 1.20%, *N* = 3 for Cas9–Klenow, *P* = 0.2509).

Analyzing Cas9- and Cas9–Klenow-induced mutation profiles at the *HBB* ‘off-target’ site found that fusing Klenow fragment to Cas9 also increased 1-bp deletions and decreased >1-bp deletions, as observed at the *HBB* on-target site (Supplementary Figure S8). In addition, TIS at this ‘off-target’ was also not increased by fusing Klenow fragment to Cas9, similar to that observed at the *HBB* on-target site. Considering that *HBB*/HEK293T was the only locus/cell where fusing Klenow fragment to Cas9 did not increase TIS in eight loci/cells examined (Table 1), our observation does not necessary indicate that fusing Klenow fragment to Cas9 will not increase TIS at off-targets. On the contrary, whether fusion will increase TIS depends on the sequence, the chromatin environment and the cell type of the off-targets.

Altogether, these experiments demonstrate that targeting the Klenow fragment to DSBs did not increase DNA mutation at DSBs or overall off-targets.

## DISCUSSION

Here, we report that targeting *E. coli* DNA pol I or the Klenow fragment to DSBs through making Cas9 fusion proteins increased the ratio of small deletions versus large deletions and the ratio of TIS versus non-TIS. These effects were observed in eight loci/cells, involving four cell types (one cell line and three primary cell types) and five target sites. We conclude that the effects of reducing deletion sizes and increasing TIS over non-TIS are not cell type or target site specific. In primary cells, fusing the Klenow fragment to Cas9 caused a significant increase in overall INDEL rates in four of five cases. Importantly, doing so suppressed the generation of on-target deletions >500 bp.

DNA resection is necessary for the HDR, MMEJ and SSA pathways. The latter two alternative NHEJ DNA repair pathways will generate short and long deletions. The MRE11–RAD50–NBS1 complex is responsible for initiating DNA resection, and EXO1, BLM and DNA2 are responsible for extensive resection (7,16,19,20). We attempted to suppress the generation of large deletions via counteracting DNA resection. Our data suggest that interfering with DNA resection is one of the mechanisms by which the Klenow fragment affects deletion sizes. First, we found that knocking down CtIP, protein involved in DNA resection (7,16,19,20), increased 1-bp deletions in IMR90 cells. Second, the effects of the Klenow fragment on deletion sizes were lost under CtIP knockdown in HEK293T cells. Our observations are consistent with a recent report that inhibiting the MRE11 complex causes the suppression of MMEJ (54). We noted that a frequently observed 11-bp deletion in *CLCN5* was not affected by CtIP knockdown, but was suppressed by Cas9–Klenow fusion, suggesting that Klenow fusion also decreases resection-independent deletions. Fusing the Klenow fragment to Cas9 did not decrease the frequency of a 9-bp deletion when targeting the *DMD* exon 53 in HEK293T cells, for which the reason is unknown.

We found that pol^D705A^ and Klenow^D705A^ with inactivated polymerase activity had similar effects on deletion sizes to pol I and the Klenow fragment. We postulate that these proteins possibly interfered with DNA resection via the following two nonexclusive mechanisms: (i) the addition of a bulk peptide (≥629 amino acids) to the C-terminus of Cas9 may prevent the recruitment of the DNA resection complex or regulatory proteins and (ii) the residual DNA binding activity of pol^D705A^ or Klenow^D705A^ may interfere with the DNA resection. Since pol^D705A^ and Klenow^D705A^ do not affect the percentage of TIS, they may be useful when one only needs to increase small deletions and decrease large deletions. Although we propose that counteracting DNA resection is one of the mechanisms underlying the observations, interference with other cellular DNA damage repair machineries may also be responsible.

The ability of Cas9–pol and Cas9–Klenow to increase the TIS/non-TIS ratio depended on local availability of the polymerase activity. This is consistent with the observations that TISs result from filling in Cas9-generated 5′ overhangs by polymerase (6). We noted that the Klenow fragment was more active than pol I in increasing 2- and 3-bp TISs (Supplementary Figure S3). This may suggest that the 5′ exonuclease domain of pol I (absent in the Klenow fragment) could compete with the polymerase domain for 5′ overhangs: the former removes 5′ overhangs and favors deletions, whereas the latter fills in the 5′ overhang to produce TIS. Removing 1-nt 5′ overhangs could also be one of the mechanisms for pol^D705A^ to increase 1-bp deletions.

Cas9–pol and Cas9–Klenow treatment increased TIS but not non-TIS. However, when targeting *CLCN5* or intragenic site 1 in IMR90 cells, fusing the Klenow fragment to Cas9 greatly decreased overall insertions, but only the non-TIS was decreased. In human cells, DNA polymerase λ is necessary for generating both TIS and non-TIS (54). How targeting the Klenow fragment to Cas9 increased only TIS and not non-TIS (or, in some cases, only decreased non-TIS but not TIS) needs more research. Fusing pol or the Klenow fragment to Cas9 promotes filling in 5′ overhangs but may inhibit recruiting of proteins responsible for generating non-TIS.

Cas9–Klenow treatment significantly increased overall INDEL rates in four of five cases in human primary cells but not in HEK293T cells. This could be explained by several nonexclusive mechanisms: (i) counteracting DNA resection and inhibiting homologous recombination, which perfectly repairs the DNA; (ii) filling in 5′ overhangs before DNA ligase ligates the complementary 5′ overhangs without generating INDELs; or (iii) Cas9 induces p53-mediated DNA damage stress in primary cells but not in HEK293T cells (59,60), and fusing the Klenow fragment to Cas9 reduces the stress by preventing repeated futile editing. We did not examine the effects of fusing the Klenow fragment to Cas9 on HDR. We expect that it may inhibit that process, considering its effects on the DNA resection necessary for such repair. While more work is needed on this topic, targeting the Klenow domain to DSBs could be useful in genome editing applications that do not depend on HDR.

Our strategy can be used to improve the safety and efficiency of gene editing in both *in vitro* and *in vivo* applications. Fusing the Klenow domain to Cas9 decreased the generation of unpredictable large on-target DNA deletions, which have been observed by multiple groups (21,27–30). It increased genome editing efficiency in primary cells and did not increase DNA substitution rates or off-target rates. In addition, its effects on 1-bp deletions and TIS can be used to increase the percentage of desirable types of mutations to improve the efficiency of disrupting disease-causing genes or restoring disrupted genes by reframing.

## DATA AVAILABILITY

The targeted NGS data and SMRT (PacBio) sequencing data generated in this study were submitted to SRA with accession numbers PRJNA758982 and PRJNA758988, respectively.

## Supplementary Material

gkac186_Supplemental_File
