# Supplementary material for: Targeting DNA polymerase to DNA double-strand breaks reduces DNA deletion size and increases templated insertions generated by CRISPR/Cas9
Source: Nucleic Acids Res. 2022 Mar 22;50(7):3944–57. doi: 10.1093/nar/gkac186 (PMC9023269; doi:10.1093/nar/gkac186)
Supplement: gkac186_Supplemental_File [file gkac186_supplemental_file.pdf]

Targeting DNA Polymerase to DNA double-strand breaks reduces DNA deletion size and increases templated insertions generated by CRISPR/Cas9

Kyung Yoo[1], Manish Kumar Yadav[1], Qianqian Song[2], Anthony Atala[1], Baisong Lu[1]*

[1]Wake Forest Institute for Regenerative Medicine, Wake Forest University Health Sciences, Winston-Salem, North Carolina, 27101, United States of America

[2]Department of Cancer Biology, Wake Forest University Health Sciences, Winston-Salem, NC, 27157

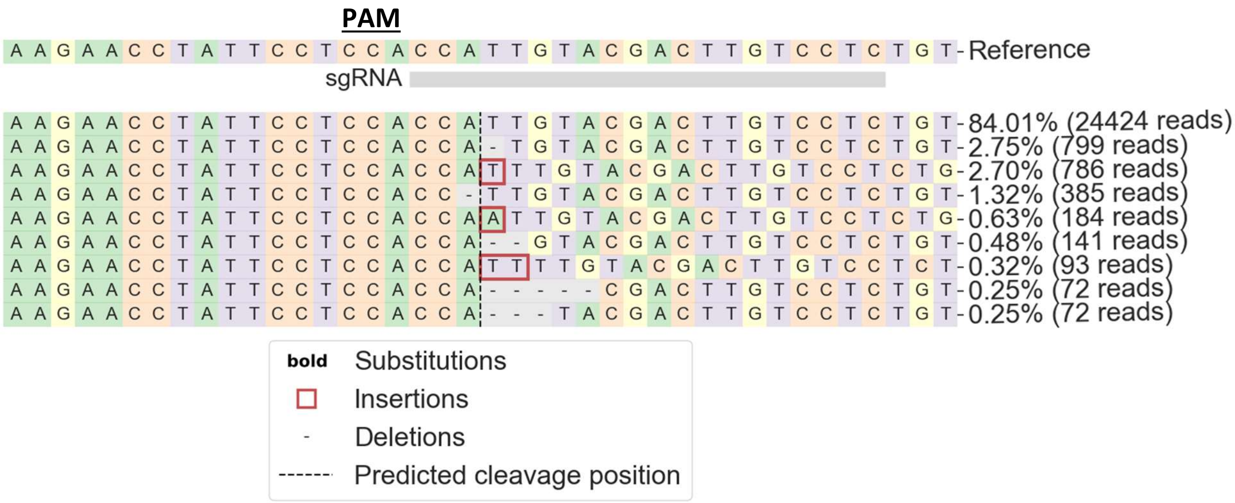

Supplementary Figure S1. NGS analysis of the integrated target sequence in GFP-reporter cells treated with *CLCN5* sgRNA and Cas9-pol. Data were analyzed by CRISPResso2.

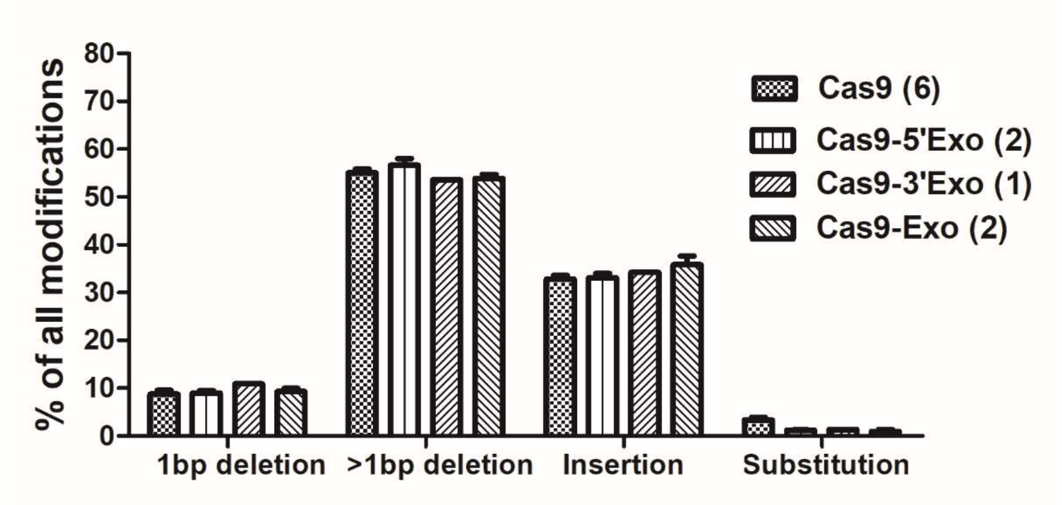

Supplementary Figure S2. Cas9 and various exonuclease fusions had similar mutation profiles as Cas9. Numbers in parentheses indicate replicate numbers. Data were analyzed by CRISPResso2.

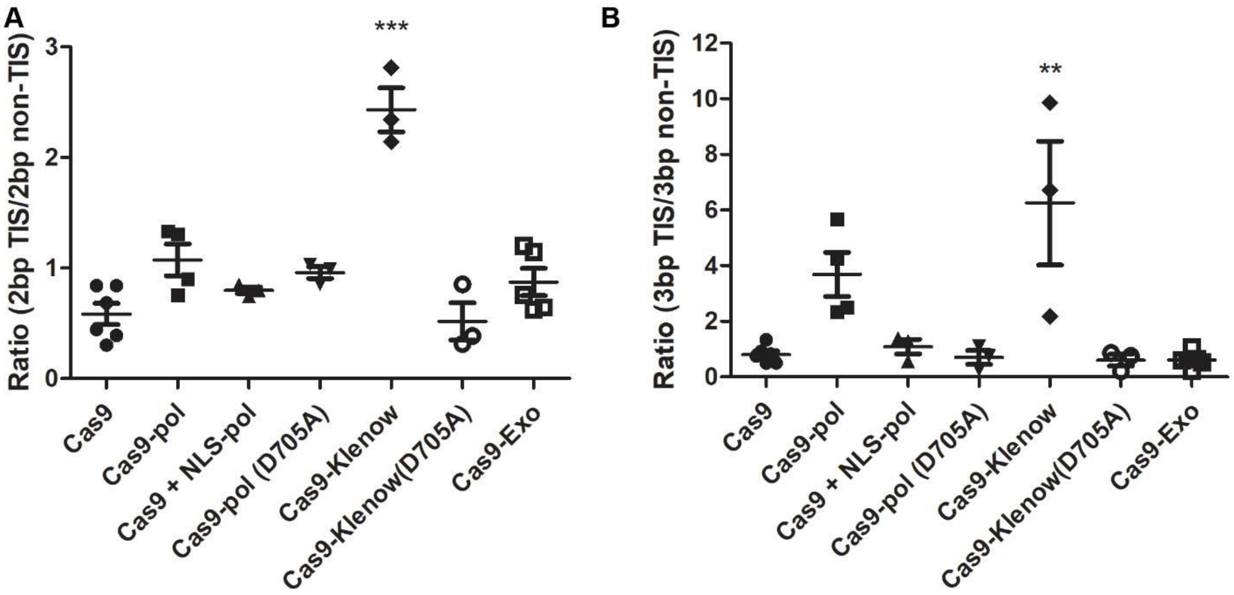

Supplementary Figure S3. Effects of different pol I domains and mutants on 2-bp (A) and 3-bp (B) TIS. The data for exo, 3' exo and 5' exo fusion proteins targeting *CLCN5* in HEK293T cells were pooled into one group. Each dot indicates one datum point. ** and *** indicate $p<0.05$ and $p<0.001$ compared with Cas9 group. Tukey's Multiple Comparison Test was performed following one-way ANOVA. Cas9-pol showed a trend of increase but did not reach statistical significance due to large intra group variation.

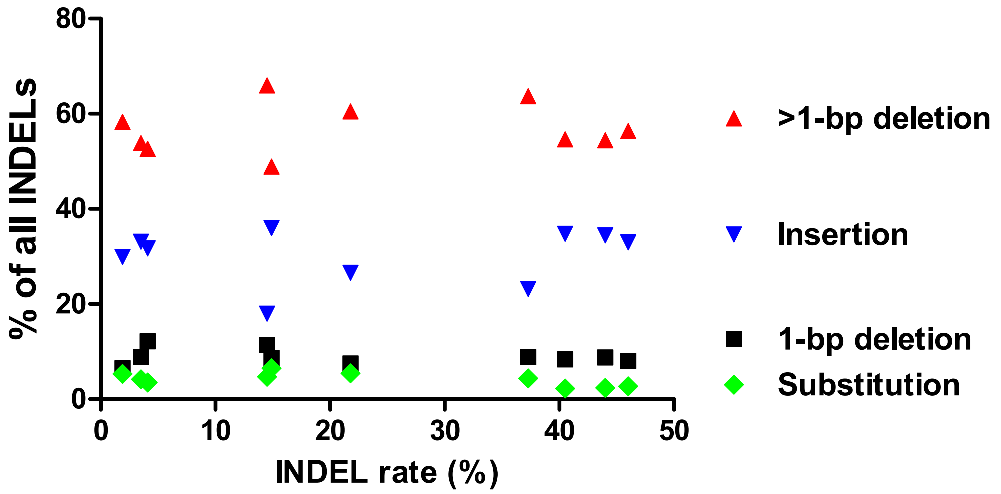

Supplementary Figure S4. No effects of overall INDEL rates on the percentages of different mutation types. Data were analyzed by CRISPResso2.

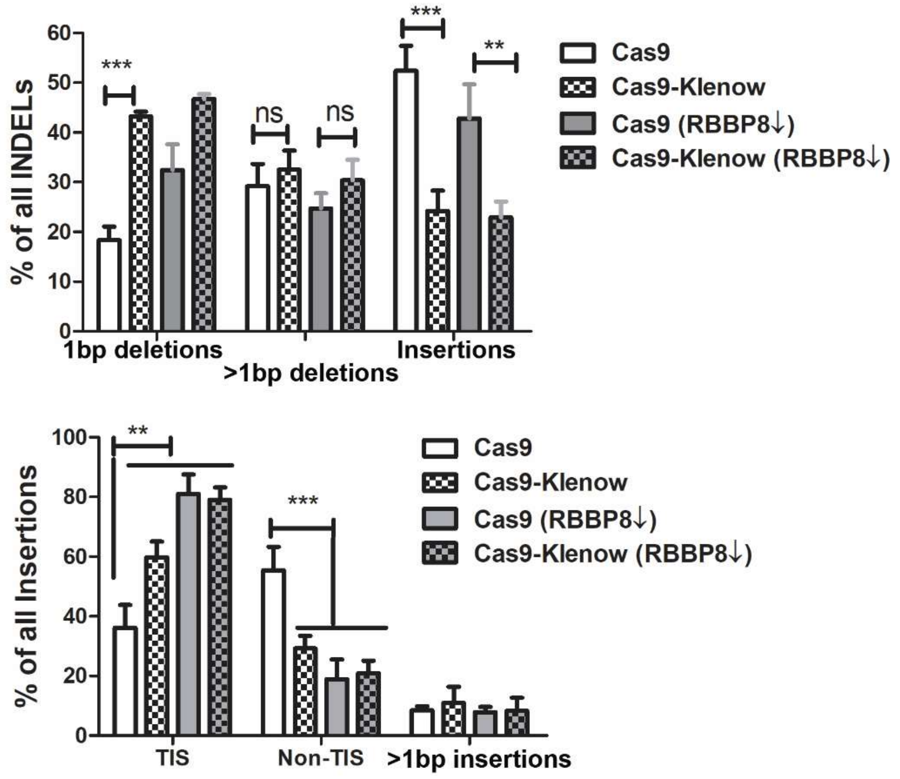

Supplementary Figure S5. Effects of *RBBP8* Knockdown on *CLCN5* mutation profiles in IMR90 cells. Top image: effects on deletions and overall insertions. Bottom image: Effects on 1-bp TIS and 1-bp non-TIS. Each group had 3 biological replicates. Two-way ANOVA was

followed by Bonferroni posttests. ** and *** indicate p<0.01 and p<0.001 between the indicated groups.

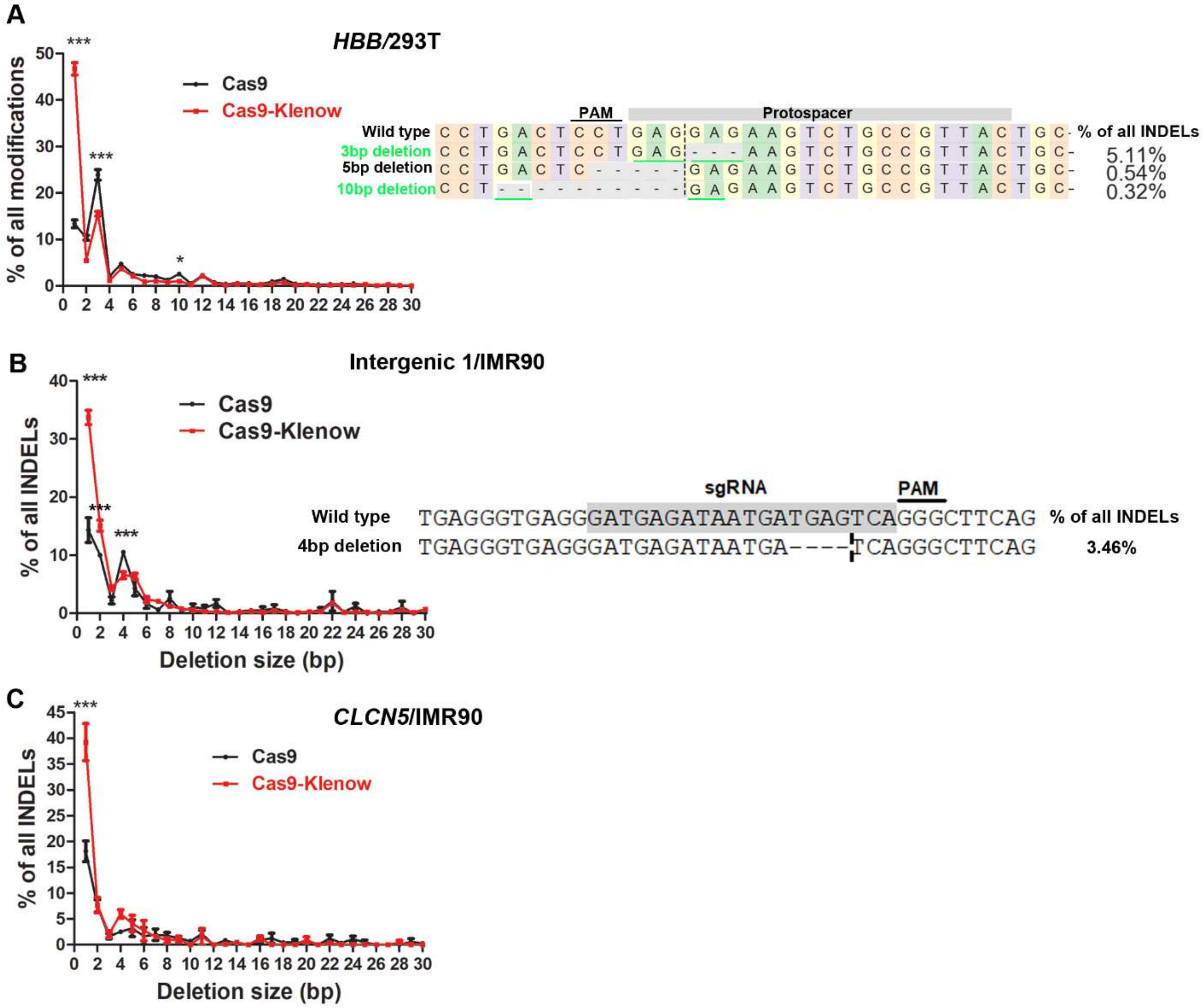

Supplementary Figure S6. Deletions decreased by Cas9-Klenow and their sequences. A. Targeting *HBB* in HEK293T cells. B. Targeting Intergenic 1 in IMR90 cells. C. Targeting *CLCN5* in IMR90 cells. The left images show the percentages of deletion sizes. The right images show the sequences of the most commonly observed deletions. No sequence could be listed for *CLCN5/*IMR90 since no major deletion peaks were observed. The regions underlined with green lines indicate microhomology at the predicted cleavage site (vertical dashed lines). Two-way ANOVA was followed by Bonferroni posttests. * and *** indicates p<0.05 and P<0.001 between the two groups.

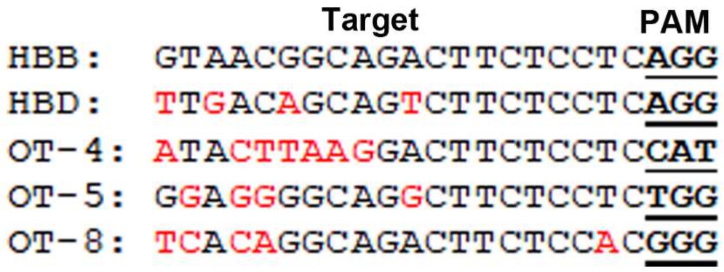

Supplementary Figure S7. Sequences of off-targets examined. PAMs are underline. Red nucleotides are those of mismatch.

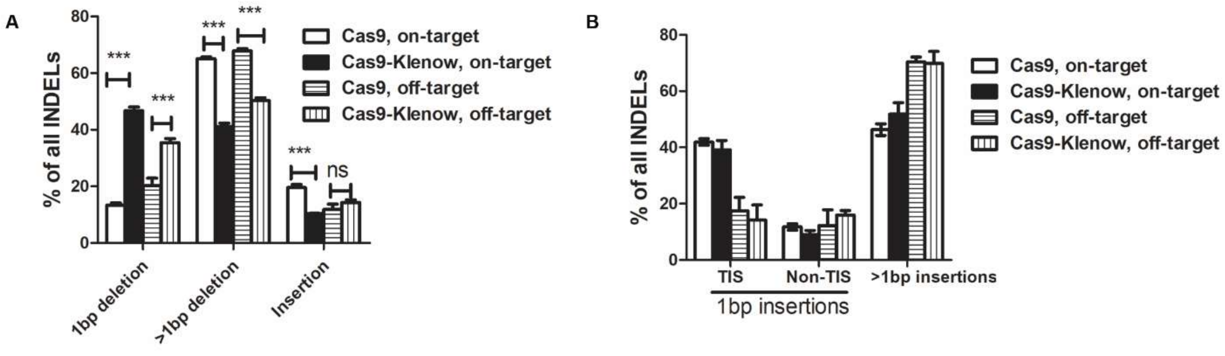

Supplementary Figure S8. Effects of fusing Klenow fragment to Cas9 on the *HBB* on-target and *HBB* "off-target" site in HEK293T cells. **A**. Deletions and insertions. **B**. TIS and non-TIS. *** indicates P<0.001 between the two groups (Bonferroni posttests following two-way ANOVA).

Supplementary Table S1. Constructs

No.	Name	Purpose	Generation strategy
1	pspCas9-POLI-Tetra-com vector	Vector plasmid for expressing spCas9-POLI fusion protein and sgRNA. There are two copies of HBB 3' UTR in the 3'UTR of spCas9-POLI to enhance expression. The Tetraloop of the sgRNA scaffold was replaced by com-aptamer to enable Cas9 RNP encapsulation into viral capsids via com-COM interaction.	The DNA sequence of E. coli DNA POLI (encoded by POLA) was optimized for human and mouse expression as "GGATCCTCTGAGACACCAGGCACCTCCGAGTCTGCCACCCCTGA<br>GGGAGGCAGCGGAGGCAGCGGCTCCGTGCAGATCCCACAGAAC<br>CCCCTGATCCTGGTGGACGGCAGCTCCTACCTGTATCGGGCCTAC<br>CACGCCTTCCCACCTCTGACAAACTCCGCCGGAGAGCCAACCGG<br>AGCCATGTATGGCGTGCTGAATATGCTGAGGAGCCTGATCATGCA<br>GTACAAGCCTACACACGCCGCCGTGGTGTTTGATGCCAAGGGCAA<br>GACCTTCCGCGACGAGCTGTTTGAGCACTACAAGAGCCACAGGC<br>CACCAATGCCTGACGATCTGAGGGCACAGATCGAGCCACTGCAC<br>GCAATGGTGAAGGCCATGGGCCTGCCTCTGCTGGCCGTGAGCGG<br>AGTGGAGGCCGACGATGTGATCGGCACACTGGCAAGGGAGGCAG<br>AGAAGGCAGGCCGCCCAGTGCTGATCTCCACCGGCGACAAGGAT<br>ATGGCCCAGCTGGTGACACCAAACATCACCCTGATCAACACCATG<br>ACAAATACCATCCTGGGCCCCGAGGAGGTGGTGAATAAGTATGGC<br>GTGCCTCCAGAGCTGATCATCGATTTCCTGGCCCTGATGGGCGAC<br>TCTAGCGATAACATCCCTGGAGTGCCAGGAGTGGGAGAAAAGAC<br>CGCACAGGCCCTGCTGCAGGGCCTGGGAGGCCTGGACACCCTGT<br>ACGCCGAGCCAGAGAAGATCGCCGGCCTGTCCTTTAGGGGCGCC<br>AAGACAATGGCCGCCAAGCTGGAGCAGAATAAGGAGGTGGCCTA<br>CCTGTCTTATCAGCTGGCCACAATCAAGACCGACGTGGAGCTGGA<br>GCTGACCTGCGAGCAGCTGGAGGTGCAGCAGCCTGCAGCAGAGG<br>AGCTGCTGGGCCTGTTCAAGAAGTACGAGTTTAAGAGATGGACAG<br>CCGATGTGGAGGCCGGCAAGTGGCTGCAGGCAAAGGGAGCAAA<br>GCCAGCAGCAAAGCCACAGGAGACAAGCGTGGCAGACGAGGCAC

No.	Name	Purpose	Generation strategy
			CAGAGGTGACAGCCACCGTGATCTCCTACGATAACTATGTGACAA<br>TCCTGGACGAGGAGACACTGAAGGCCTGGATCGCCAAGCTGGAG<br>AAGGCCCCCGTGTTCGCCTTTGATACAGAGACAGACAGCCTGGAT<br>AACATCTCCGCCAATCTGGTGGGCCTGTCTTTCGCCATCGAGCCT<br>GGCGTGGCCGCCTATATCCCAGTGGCCCACGACTACCTGGATGC<br>CCCCGACCAGATCAGCAGGGAGAGAGCCCTGGAGCTGCTGAAGC<br>CTCTGCTGGAGGATGAGAAGGCCCTGAAGGTCGGCCAGAACCTG<br>AAGTATGACAGGGGCATCCTGGCCAATTACGGCATCGAGCTGAGA<br>GGCATCGCCTTTGACACCATGCTGGAGTCTTATATCCTGAATAGC<br>GTGGCAGGCCGGCACGACATGGATTCCCTGGCCGAGAGGTGGCT<br>GAAGCACAAGACAATCACCTTCGAGGAGATCGCCGGCAAGGGCA<br>AGAACCAGCTGACCTTCAACCAGATCGCCCTGGAGGAGGCAGGC<br>AGGTACGCAGCAGAGGACGCAGATGTGACCCTGCAGCTGCACCT<br>GAAGATGTGGCCAGATCTGCAGAAGCACAAGGGCCCCCTGAACG<br>TGTTCGAGAATATCGAGATGCCCCTGGTGCCTGTGCTGAGCCGGA<br>TCGAGCGCAACGGCGTGAAGATCGACCCTAAGGTGCTGCACAAT<br>CACTCCGAGGAGCTGACCCTGAGACTGGCCGAGCTGGAGAAGAA<br>GGCCCACGAGATCGCCGGCGAGGAGTTCAACCTGTCCTCTACAA<br>AGCAGCTGCAGACCATCCTGTTTGAGAAGCAGGGCATCAAGCCCC<br>TGAAGAAAACCCCTGGAGGAGCACCATCTACCAGCGAGGAGGTG<br>CTGGAGGAGCTGGCCCTGGATTATCCCCTGCCTAAAGTGATCCTG<br>GAGTACCGGGGCCTGGCCAAGCTGAAGTCTACATATACCGACAAG<br>CTGCCCCTGATGATCAACCCTAAGACAGGAAGGGTGCACACCAGC<br>TACCACCAGGCAGTGACAGCAACCGGCCGCCTGAGCTCCACCGA<br>TCCAAACCTGCAGAATATCCCCGTGAGGAATGAGGAGGGCAGGA<br>GAATCAGACAGGCCTTCATCGCCCCCGAGGATTATGTGATCGTGT<br>CCGCCGACTACTCTCAGATCGAGCTGAGGATCATGGCCCACCTGT<br>CCAGAGATAAGGGCCTGCTGACAGCCTTCGCCGAGGGCAAGGAC<br>ATCCACAGGGCAACCGCAGCAGAGGTGTTTGGCCTGCCTCTGGA<br>GACAGTGACCTCCGAGCAGCGGCGCTCTGCCAAGGCCATCAACT<br>TCGGCCTGATCTATGGCATGTCTGCCTTTGGCCTGGCCAGGCAGC<br>TGAATATCCCTAGAAAGGAGGCCCAGAAGTACATGGACCTGTATT<br>TCGAGAGGTACCCAGGCGTGCTGGAGTACATGGAGAGGACAAGG<br>GCACAGGCAAAGGAGCAGGGCTATGTGGAGACACTGGATGGCAG<br>GAGACTGTACCTGCCAGACATCAAGTCTAGCAACGGAGCAAGGA<br>GGGCAGCAGCAGAGAGGGCCGCCATCAATGCCCCCATGCAGGG<br>CACAGCCGCCGATATCATCAAGAGAGCCATGATCGCAGTGGACG<br>CATGGCTGCAGGCAGAGCAGCCAAGGGTGAGAATGATCATGCAG<br>GTGCACGATGAGCTGGTGTTTGAGGTGCACAAGGACGATGTGGA<br>CGCCGTGGCCAAGCAGATCCACCAGCTGATGGAGAACTGTACCA<br>GGCTGGATGTGCCACTGCTGGTGGAAGTGGGCAGCGGAGAGAAT<br>TGGGACCAGGCCCACTGAGGATCC" and was inserted into BamH1 site of pspCas9-Tetra-com vector. The peptide sequence is:<br>MDYKDHDGDYKDHDIDYKDDDDKMAPKKKRKVGIHGVPAADKKYSI<br>GLDIGTNSVGWAVITDEYKVPSKKFKVLGNTDRHSIKKNLIGALLFDS<br>GETAEATRLKRTARRRYTRRKNRICYLQEIFSNEMAKVDDSFFHRLE<br>ESFLVEEDKKHERHPIFGNIVDEVAYHEKYPTIYHLRKKLVDSTDKADL<br>RLIYLALAHMIKFRGHFLIEGDLNPDNSDVDKLFIQLVQTYNQLFEENPI<br>NASGVDAKAILSARLSKSRRLENLIAQLPGEKKNGLFGNLIALSLGLTP<br>NFKSNFDLAEDAKLQLSKDTYDDDLDNLLAQIGDQYADLFLAAKNLSD<br>AILLSDILRVNTEITKAPLSASMIKRYDEHHQDLTLLKALVRQQLPEKYK<br>EIFFDQSKNGYAGYIDGGASQEEFYKFIKPILEKMDGTEELLVKLNRE<br>DLLRKQRTFDNGSIPHQIHLGELHAILRRQEDFYPFLKDNREKIEKILTF<br>RIPYYVGPLARGNSRFAWMTRKSEETITPWNFEEVVDKGASAQSFIE<br>RMTNFDKNLPNEKVLPKHSLLYEYFTVYNELTKVKYVTEGMRKPAFL<br>SGEQKKAIVDLLFKTNRKVTVKQLKEDYFKKIECFDSVEISGVEDRFN<br>ASLGTYHDLLKIIKDKDFLDNEENEDILEDIVLTLTLFEDREMIEERLKTY<br>AHLFDDKVMKQLKRRRYTGWGRLSRKLINGIRDKQSGKTILDFLKSD<br>GFANRNFMQLIHDDSLTFKEDIQKAQVSGQGDSLHEHIANLAGSPAIK<br>KGILQTVKVVDELVKVMGRHKPENIVIEMARENQTTQKGQKNSRERM<br>KRIEEGIKELGSQILKEHPVENTQLQNEKLYLYYLQNGRDMYVDQELD<br>INRLSDYDVDHIVPQSFLKDDSIDNKVLTRSDKNRGKSDNVPSEEVVK<br>KMKNYWRQLLNAKLITQRKFDNLTKAERGGLSELDKAGFIKRQLVET<br>RQITKHVAQILDSRMNTKYDENDKLIREVKVITLKSKLVSDFRKDFQFY<br>KVREINNYHHAHDAYLNAVVGTALIKKYPKLESEFVYGDYKVYDVRK<br>MIAKSEQEIGKATAKYFFYSNIMNFFKTEITLANGEIRKRPLIETNGETG<br>EIVWDKGRDFATVRKVLSMPQVNIVKKTEVQTGGFSKESILPKRNSD<br>KLIARKKDWDPKKYGGFDSPTVAYSVLVVAKVEKGKSKKLKSVKELL

No.	Name	Purpose	Generation strategy
			GITIMERSSFEKNPIDFLEAKGYKEVKKDLIIKLPKYSLFELENGRKRML<br>ASAGELQKGNELALPSKYVNFLYLASHYEKLKGSPEDNEQKQLFVEQ<br>HKHYLDEIIEQISEFSKRVILADANLDKVLSAYNKHRDKPIREQAENIIHL<br>FTLTNLGAPAAFKYFDTTIDRKRYTSTKEVLDATLIHQSITGLYETRIDL<br>SQLGGDKRPAATKKAGQAKKKKGSSETPGTSESATPEGGSGGSGS<br>VQIPQNPLILVDGSSYLYRAYHAFPPLTNSAGEPTGAMYGVLNMLRSL<br>IMQYKPTHAAVVFDAKGKTFRDELFEHYKSHRPPMPDDLRAQIEPLH<br>AMVKAMGLPLLAVSGVEADDVIGTLAREAEKAGRPVLISTGDKDMAQ<br>LVTPNITLINTMTNTILGPEEVVNKYGVPPELIIDFLALMGDSSDNIPGV<br>PGVGEKTAQALLQGLGGLDTLYAEPEKIAGLSFRGAKTMAAKLEQNK<br>EVAYLSYQLATIKTDVELELTCEQLEVQQPAAEELLGLFKKYEFKRWT<br>ADVEAGKWLQAKGAKPAAKPQETSVADEAPEVTATVISYDNYVTILD<br>EETLKAWIAKLEKAPVFAFDTETDSLDNISANLVGLSFAIEPGVAAYIPV<br>AHDYLDAPDQISRERALELLKPLLEDEKALKVGQNLKYDRGILANYGIE<br>LRGIAFDTMLESYILNSVAGRHDMDSLAERWLKHKTITFEEIAGKGKN<br>QLTFNQIALEEAGRYAAEDADVTLQLHLKMWPDLQKHKGPLNVFENI<br>EMPLVPVLSRIERNGVKIDPKVLHNHSEELTLRLAELEKKAHEIAGEEF<br>NLSSTKQLQTILFEKQGIKPLKKTPGGAPSTSEEVLEELALDYPLPKVIL<br>EYRGLAKLKSTYTDKLPLMINPKTGRVHTSYHQAVTATGRLSSTDPNL<br>QNIPVRNEEGRRIRQAFIAPEDYVIVSADYSQIELRIMAHLSRDKGLLT<br>AFAEGKDIHRATAAEVFGLPLETVTSEQRRSAKAINFGLIYGMSAFGL<br>ARQLNIPRKEAQKYMDLYFERYPGVLEYMERTRAQAKEQGYVETLD<br>GRRLYLPDIKSSNGARRAAAERAAINAPMQGTAADIIKRAMIAVDAWL<br>QAEQPRVRMIMQVHDELVFEVHKDDVDAVAKQIHQLMENCTRLDVP<br>LLVEVGSGENWDQAH (Color Key: Cas9-linker-Polymerase)
2	pspCas9-poli-Tetra-com-clcn5-sp-g1	Vector plasmid for expressing spCas9-polI fusion protein and sgRNA targeting human CLCN5 gene.	The annealed product of hCLCN5-sp-g1-F (ACCGGAGGACAAGTCGTACAATGG) and hCLCN5-sp-g1-R (AAACCCATTGTACGACTTGTCCTC) was inserted between the BbsI sites of pspCas9-POLI-Tetra-com vector by T4 DNA ligase.
3	pspCas9-Klenow-Tetra-com-CLCN5-sp-g1	Plasmid for expressing spCas9-Klenow fusion protein and sgRNA targeting human CLCN5 gene.	Use pspCas9-POLI-tetra-com-hCLCN5-sp-g1 as the template and Klenow-F (GTCTGCCACCCCTGAGGGAGGAGCACCAGAG GTGACAGCCAC) and Klenow-R (TTGTGCAGCACCTTAGGGTCG) as the primers to amplify a 735 bp fragment. This DNA was inserted between the two Bsu36I sites of pspCas9-POLI-tetra-com-hCLCN5-sp-g1 by Infusion reaction.<br>The end result is the removal of the DNA coding for the 5’ exonuclease activity of E. coli POLI. Nucleic acid sequence:<br>ATGGACTATAAGGACCACGACGGAGACTACAAGGATCATGATATT<br>GATTACAAAGACGATGACGATAAGATGGCCCCAAAGAAGAAGCGG<br>AAGGTCGGTATCCACGGAGTCCCAGCAGCCGACAAGAAGTACAG<br>CATCGGCCTGGACATCGGCACCAACTCTGTGGGCTGGGCCGTGA<br>TCACCGACGAGTACAAGGTGCCCAGCAAGAAATTCAAGGTGCTGG<br>GCAACACCGACCGGCACAGCATCAAGAAGAACCTGATCGGAGCC<br>CTGCTGTTCGACAGCGGCGAAACAGCCGAGGCCACCCGGCTGAA<br>GAGAACCGCCAGAAGAAGATACACCAGACGGAAGAACCGGATCT<br>GCTATCTGCAAGAGATCTTCAGCAACGAGATGGCCAAGGTGGACG<br>ACAGCTTCTTCCACAGACTGGAAGAGTCCTTCCTGGTGGAAGAGG<br>ATAAGAAGCACGAGCGGCACCCCATCTTCGGCAACATCGTGGAC<br>GAGGTGGCCTACCACGAGAAGTACCCCACCATCTACCACCTGAGA<br>AAGAAACTGGTGGACAGCACCGACAAGGCCGACCTGCGGCTGAT<br>CTATCTGGCCCTGGCCCACATGATCAAGTTCCGGGGCCACTTCCT<br>GATCGAGGGCGACCTGAACCCCGACAACAGCGACGTGGACAAGC<br>TGTTCATCCAGCTGGTGCAGACCTACAACCAGCTGTTCGAGGAAA<br>ACCCCATCAACGCCAGCGGCGTGGACGCCAAGGCCATCCTGTCT<br>GCCAGACTGAGCAAGAGCAGACGGCTGGAAAATCTGATCGCCCA<br>GCTGCCCGGCGAGAAGAAGAATGGCCTGTTCGGAAACCTGATTG

No.	Name	Purpose	Generation strategy
			CCCTGAGCCTGGGCCTGACCCCCAACTTCAAGAGCAACTTCGACC<br>TGGCCGAGGATGCCAAACTGCAGCTGAGCAAGGACACCTACGAC<br>GACGACCTGGACAACCTGCTGGCCCAGATCGGCGACCAGTACGC<br>CGACCTGTTTCTGGCCGCCAAGAACCTGTCCGACGCCATCCTGCT<br>GAGCGACATCCTGAGAGTGAACACCGAGATCACCAAGGCCCCCC<br>TGAGCGCCTCTATGATCAAGAGATACGACGAGCACCACCAGGACC<br>TGACCCTGCTGAAAGCTCTCGTGCGGCAGCAGCTGCCTGAGAAG<br>TACAAAGAGATTTTCTTCGACCAGAGCAAGAACGGCTACGCCGGC<br>TACATTGACGGCGGAGCCAGCCAGGAAGAGTTCTACAAGTTCATC<br>AAGCCCATCCTGGAAAAGATGGACGGCACCGAGGAACTGCTCGT<br>GAAGCTGAACAGAGAGGACCTGCTGCGGAAGCAGCGGACCTTCG<br>ACAACGGCAGCATCCCCCACCAGATCCACCTGGGAGAGCTGCAC<br>GCCATTCTGCGGCGGCAGGAAGATTTTTACCCATTCCTGAAGGAC<br>AACCGGGAAAAGATCGAGAAGATCCTGACCTTCCGCATCCCCTAC<br>TACGTGGGCCCTCTGGCCAGGGGAAACAGCAGATTCGCCTGGAT<br>GACCAGAAAGAGCGAGGAAACCATCACCCCCTGGAACTTCGAGG<br>AAGTGGTGGACAAGGGCGCTTCCGCCCAGAGCTTCATCGAGCGG<br>ATGACCAACTTCGATAAGAACCTGCCCAACGAGAAGGTGCTGCCC<br>AAGCACAGCCTGCTGTACGAGTACTTCACCGTGTATAACGAGCTG<br>ACCAAAGTGAAATACGTGACCGAGGGAATGAGAAAGCCCGCCTTC<br>CTGAGCGGCGAGCAGAAAAAGGCCATCGTGGACCTGCTGTTCAA<br>GACCAACCGGAAAGTGACCGTGAAGCAGCTGAAAGAGGACTACTT<br>CAAGAAAATCGAGTGCTTCGACTCCGTGGAAATCTCCGGCGTGGA<br>AGATCGGTTCAACGCCTCCCTGGGCACATACCACGATCTGCTGAA<br>AATTATCAAGGACAAGGACTTCCTGGACAATGAGGAAAACGAGGA<br>CATTCTGGAAGATATCGTGCTGACCCTGACACTGTTTGAGGACAG<br>AGAGATGATCGAGGAACGGCTGAAAACCTATGCCCACCTGTTCGA<br>CGACAAAGTGATGAAGCAGCTGAAGCGGCGGAGATACACCGGCT<br>GGGGCAGGCTGAGCCGGAAGCTGATCAACGGCATCCGGGACAA<br>GCAGTCCGGCAAGACAATCCTGGATTTCCTGAAGTCCGACGGCTT<br>CGCCAACAGAAACTTCATGCAGCTGATCCACGACGACAGCCTGAC<br>CTTTAAAGAGGACATCCAGAAAGCCCAGGTGTCCGGCCAGGGCG<br>ATAGCCTGCACGAGCACATTGCCAATCTGGCCGGCAGCCCCGCC<br>ATTAAGAAGGGCATCCTGCAGACAGTGAAGGTGGTGGACGAGCT<br>CGTGAAAGTGATGGGCCGGCACAAGCCCGAGAACATCGTGATCG<br>AAATGGCCAGAGAGAACCAGACCACCCAGAAGGGACAGAAGAAC<br>AGCCGCGAGAGAATGAAGCGGATCGAAGAGGGCATCAAAGAGCT<br>GGGCAGCCAGATCCTGAAAGAACACCCCGTGGAAAACACCCAGC<br>TGCAGAACGAGAAGCTGTACCTGTACTACCTGCAGAATGGGCGG<br>GATATGTACGTGGACCAGGAACTGGACATCAACCGGCTGTCCGAC<br>TACGATGTGGACCATATCGTGCCTCAGAGCTTTCTGAAGGACGAC<br>TCCATCGACAACAAGGTGCTGACCAGAAGCGACAAGAACCGGGG<br>CAAGAGCGACAACGTGCCCTCCGAAGAGGTCGTGAAGAAGATGA<br>AGAACTACTGGCGGCAGCTGCTGAACGCCAAGCTGATTACCCAGA<br>GAAAGTTCGACAATCTGACCAAGGCCGAGAGAGGCGGCCTGAGC<br>GAACTGGATAAGGCCGGCTTCATCAAGAGACAGCTGGTGGAAAC<br>CCGGCAGATCACAAAGCACGTGGCACAGATCCTGGACTCCCGGA<br>TGAACACTAAGTACGACGAGAATGACAAGCTGATCCGGGAAGTGA<br>AAGTGATCACCCTGAAGTCCAAGCTGGTGTCCGATTTCCGGAAGG<br>ATTTCCAGTTTTACAAAGTGCGCGAGATCAACAACTACCACCACGC<br>CCACGACGCCTACCTGAACGCCGTCGTGGGAACCGCCCTGATCA<br>AAAAGTACCCTAAGCTGGAAAGCGAGTTCGTGTACGGCGACTACA<br>AGGTGTACGACGTGCGGAAGATGATCGCCAAGAGCGAGCAGGAA<br>ATCGGCAAGGCTACCGCCAAGTACTTCTTCTACAGCAACATCATG<br>AACTTTTTCAAGACCGAGATTACCCTGGCCAACGGCGAGATCCGG<br>AAGCGGCCTCTGATCGAGACAAACGGCGAAACCGGGGAGATCGT<br>GTGGGATAAGGGCCGGGATTTTGCCACCGTGCGGAAAGTGCTGA<br>GCATGCCCCAAGTGAATATCGTGAAAAAGACCGAGGTGCAGACAG<br>GCGGCTTCAGCAAAGAGTCTATCCTGCCCAAGAGGAACAGCGATA<br>AGCTGATCGCCAGAAAGAAGGACTGGGACCCTAAGAAGTACGGC<br>GGCTTCGACAGCCCCACCGTGGCCTATTCTGTGCTGGTGGTGGC<br>CAAAGTGGAAAAGGGCAAGTCCAAGAAACTGAAGAGTGTGAAAGA<br>GCTGCTGGGGATCACCATCATGGAAAGAAGCAGCTTCGAGAAGAA<br>TCCCATCGACTTTCTGGAAGCCAAGGGCTACAAAGAAGTGAAAAA<br>GGACCTGATCATCAAGCTGCCTAAGTACTCCCTGTTCGAGCTGGA<br>AAACGGCCGGAAGAGAATGCTGGCCTCTGCCGGCGAACTGCAGA<br>AGGGAAACGAACTGGCCCTGCCCTCCAAATATGTGAACTTCCTGT<br>ACCTGGCCAGCCACTATGAGAAGCTGAAGGGCTCCCCCGAGGAT

No.	Name	Purpose	Generation strategy
			AATGAGCAGAAACAGCTGTTTGTGGAACAGCACAAGCACTACCTG<br>GACGAGATCATCGAGCAGATCAGCGAGTTCTCCAAGAGAGTGATC<br>CTGGCCGACGCTAATCTGGACAAAGTGCTGTCCGCCTACAACAAG<br>CACCGGGATAAGCCCATCAGAGAGCAGGCCGAGAATATCATCCA<br>CCTGTTTACCCTGACCAATCTGGGAGCCCCTGCCGCCTTCAAGTA<br>CTTTGACACCACCATCGACCGGAAGAGGTACACCAGCACCAAAGA<br>GGTGCTGGACGCCACCCTGATCCACCAGAGCATCACCGGCCTGT<br>ACGAGACACGGATCGACCTGTCTCAGCTGGGAGGCGACAAAAGG<br>CCGGCGGCCACGAAAAAGGCCGGccaggcaaaaaagaaaaaggGATCC<br>TCTGAGACACCAGGCACCTCCGAGTCTGCCACCCCTGAGGGAGG<br>AGCACCAGAGGTGACAGCCACCGTGATCTCCTACGATAACTATGT<br>GACAATCCTGGACGAGGAGACACTGAAGGCCTGGATCGCCAAGC<br>TGGAGAAGGCCCCCGTGTTCGCCTTTGATACAGAGACAGACAGCC<br>TGGATAACATCTCCGCCAATCTGGTGGGCCTGTCTTTCGCCATCG<br>AGCCTGGCGTGGCCGCCTATATCCCAGTGGCCCACGACTACCTG<br>GATGCCCCCGACCAGATCAGCAGGGAGAGAGCCCTGGAGCTGCT<br>GAAGCCTCTGCTGGAGGATGAGAAGGCCCTGAAGGTCGGCCAGA<br>ACCTGAAGTATGACAGGGGCATCCTGGCCAATTACGGCATCGAGC<br>TGAGAGGCATCGCCTTTGACACCATGCTGGAGTCTTATATCCTGA<br>ATAGCGTGGCAGGCCGGCACGACATGGATTCCCTGGCCGAGAGG<br>TGGCTGAAGCACAAGACAATCACCTTCGAGGAGATCGCCGGCAA<br>GGGCAAGAACCAGCTGACCTTCAACCAGATCGCCCTGGAGGAGG<br>CAGGCAGGTACGCAGCAGAGGACGCAGATGTGACCCTGCAGCTG<br>CACCTGAAGATGTGGCCAGATCTGCAGAAGCACAAGGGCCCCCT<br>GAACGTCTTTGAGAATATCGAGATGCCCCTGGTGCCTGTGCTGAG<br>CCGGATCGAGCGCAACGGCGTGAAGATCGACCCTAAGGTGCTGC<br>ACAATCACTCCGAGGAGCTGACCCTGAGACTGGCCGAGCTGGAG<br>AAGAAGGCCCACGAGATCGCCGGCGAGGAGTTCAACCTGTCCTC<br>TACAAAGCAGCTGCAGACCATCCTGTTTGAGAAGCAGGGCATCAA<br>GCCCCTGAAGAAAACCCCTGGAGGAGCACCATCTACCAGCGAGG<br>AGGTGCTGGAGGAGCTGGCCCTGGATTATCCCCTGCCTAAAGTGA<br>TCCTGGAGTACCGGGGCCTGGCCAAGCTGAAGTCTACATATACCG<br>ACAAGCTGCCCCTGATGATCAACCCTAAGACAGGCCGGGTGCACA<br>CCAGCTACCACCAGGCAGTGACAGCAACCGGCCGCCTGAGCTCC<br>ACCGATCCAAACCTGCAGAATATCCCCGTGAGGAATGAGGAGGG<br>CAGGAGAATCAGACAGGCCTTCATCGCCCCCGAGGATTATGTGAT<br>CGTGTCCGCCGACTACTCTCAGATCGAGCTGAGGATCATGGCCCA<br>CCTGTCCAGAGATAAGGGCCTGCTGACAGCCTTCGCCGAGGGCA<br>AGGACATCCACAGGGCAACCGCAGCAGAGGTGTTTGGCCTGCCT<br>CTGGAGACAGTGACCTCCGAGCAGCGGCGCTCTGCCAAGGCCAT<br>CAACTTCGGCCTGATCTATGGCATGTCTGCCTTTGGCCTGGCCAG<br>GCAGCTGAATATCCCTAGAAAGGAGGCCCAGAAGTACATGGACCT<br>GTATTTCGAGAGATACCCCGGCGTGCTGGAGTACATGGAGAGGA<br>CACGCGCACAGGCAAAGGAGCAGGGCTATGTGGAGACACTGGAT<br>GGCAGGAGACTGTACCTGCCAGACATCAAGTCTAGCAACGGAGC<br>AAGGAGGGCAGCAGCAGAGAGGGCCGCCATCAATGCCCCCATGC<br>AGGGCACAGCCGCCGATATCATCAAGAGAGCCATGATCGCAGTG<br>GACGCCTGGCTGCAGGCAGAGCAGCCAAGGGTGAGAATGATCAT<br>GCAGGTGCACGATGAGCTGGTGTTTGAGGTGCACAAGGACGATG<br>TGGACGCCGTGGCCAAGCAGATCCACCAGCTGATGGAGAACTGT<br>ACCCGCCTGGATGTGCCACTGCTGGTGGAAGTGGGAAGCGGAGA<br>GAATTGGGACCAGGCCCACTGAG. Peptide sequence:<br>MDYKDHDGDYKDHDIDYKDDDDKMAPKKKRKVGIHGVPAADKKYSI<br>GLDIGTNSVGWAVITDEYKVPSKKFKVLGNTDRHSIKKNLIGALLFDS<br>GETAEATRLKRTARRRYTRRKNRICYLQEIFSNEMAKVDDSFFHRLE<br>ESFLVEEDKKHERHPIFGNIVDEVAYHEKYPTIYHLRKKLVDSTDKADL<br>RLIYLALAHMIKFRGHFLIEGDLNPDNSDVDKLFIQLVQTYNQLFEENPI<br>NASGVDAKAILSARLSKSRRLENLIAQLPGEKKNGLFGNLIALSLGLTP<br>NFKSNFDLAEDAKLQLSKDTYDDDLDNLLAQIGDQYADLFLAAKNLSD<br>AILLSDILRVNTEITKAPLSASMIKRYDEHHQDLTLLKALVRQQLPEKYK<br>EIFFDQSKNGYAGYIDGGASQEEFYKFIKPILEKMDGTEELLVKLNRE<br>DLLRKQRTFDNGSIPHQIHLGELHAILRRQEDFYPFLKDNREKIEKILTF<br>RIPYYVGPLARGNSRFAWMTRKSEETITPWNFEEVVDKGASAQSFIE<br>RMTNFDKNLPNEKVLPKHSLLYEYFTVYNELTKVKYVTEGMRKPAFL<br>SGEQKKAIVDLLFKTNRKVTVKQLKEDYFKKIECFDSVEISGVEDRFN<br>ASLGTYHDLLKIIKDKDFLDNEENEDILEDIVLTLTLFEDREMIEERLKTY<br>AHLFDDKVMKQLKRRRYTGWGRLSRKLINGIRDKQSGKTILDFLKSD<br>GFANRNFMQLIHDDSLTFKEDIQKAQVSGQGDSLHEHIANLAGSPAIK

No.	Name	Purpose	Generation strategy
			KGILQTVKVVDELVKVMGRHKPENIVIEMARENQTTQKGQKNSRERM<br>KRIEEGIKELGSQILKEHPVENTQLQNEKLYLYYLQNGRDMYVDQELD<br>INRLSDYDVDHIVPQSFLKDDSIDNKVLTRSDKNRGKSDNVPSEEVVK<br>KMKNYWRQLLNAKLITQRKFDNLTKAERGGLSELDKAGFIKRQLVET<br>RQITKHVAQILDSRMNTKYDENDKLIREVKVITLKSKLVSDFRKDFQFY<br>KVREINNYHHAHDAYLNAVVGTALIKKYPKLESEFVYGDYKVYDVRK<br>MIAKSEQEIGKATAKYFFYSNIMNFFKTEITLANGEIRKRPLIETNGETG<br>EIVWDKGRDFATVRKVLSMPQVNIVKKTEVQTGGFSKESILPKRNSD<br>KLIARKKDWDPKKYGGFDSPTVAYSVLVVAKVEKGKSKKLKSVKELL<br>GITIMERSSFEKNPIDFLEAKGYKEVKKDLIIKLPKYSLFELENGRKRML<br>ASAGELQKGNELALPSKYVNFLYLASHYEKLKGSPEDNEQKQLFVEQ<br>HKHYLDEIIEQISEFSKRVILADANLDKVLSAYNKHRDKPIREQAENIIHL<br>FTLTNLGAPAAFKYFDTTIDRKRYTSTKEVLDATLIHQSITGLYETRIDL<br>SQLGGDKRPAATKKAGQAKKKKGSSETPGTSESATPEGGAPEVTAT<br>VISYDNYVTILDEETLKAWIAKLEKAPVFAFDTETDSLDNISANLVGLSF<br>AIEPGVAAYIPVAHDYLDAPDQISRERALELLKPLLEDEKALKVGQNLK<br>YDRGILANYGIELRGIAFDTMLESYILNSVAGRHDMDSLAERWLKHKTI<br>TFEEIAGKGKNQLTFNQIALEEAGRYAAEDADVTLQLHLKMWPDLQK<br>HKGPLNVFENIEMPLVPVLSRIERNGVKIDPKVLHNHSEELTLRLAELE<br>KKAHEIAGEEFNLSSTKQLQTILFEKQGIKPLKKTPGGAPSTSEEVLEE<br>LALDYPLPKVILEYRGLAKLKSTYTDKLPLMINPKTGRVHTSYHQAVTA<br>TGRLSSTDPNLQNIPVRNEEGRRIRQAFIAPEDYVIVSADYSQIELRIM<br>AHLSRDKGLLTAFAEGKDIHRATAAEVFGLPLETVTSEQRRSAKAINF<br>GLIYGMSAFGLARQLNIPRKEAQKYMDLYFERYPGVLEYMERTRAQA<br>KEQGYVETLDGRRLYLPDIKSSNGARRAAAERAAINAPMQGTAADIIK<br>RAMIAVDAWLQAEQPRVRMIMQVHDELVFEVHKDDVDAVAKQIHQL<br>MENCTRLDVPLLVEVGSGENWDQAH (Color Key: Cas9-linker-Klenow)
4	pspCas9-3exo-Tetra-com-CLCN5-sp-g1	Plasmid for expressing a fusion protein of spCas9 and the 3' exonuclease domain of POLI, and sgRNA targeting human CLCN5 gene.	Use pspCas9-exo-tetra-com-hCLCN5-sp-g1 as the template and Klenow-F (GTCTGCCACCCCTGAGGGAGGAGCACCAGAG GTGACAGCCAC) and 3exo-R (CtaggaattttctagactaGTGCTTCTGCAGATCTGGCC AC) to amplify a 650 bp fragment. This fragment was inserted between the Bsu36I and XbaI sites of pspCas9-exo-tetra-com-hCLCN5-sp-g1 Infusion reaction. Nucleic acid sequence:<br>ATGGACTATAAGGACCACGACGGAGACTACAAGGATCATGATATT<br>GATTACAAAGACGATGACGATAAGATGGCCCCAAAGAAGAAGCGG<br>AAGGTCGGTATCCACGGAGTCCCAGCAGCCGACAAGAAGTACAG<br>CATCGGCCTGGACATCGGCACCAACTCTGTGGGCTGGGCCGTGA<br>TCACCGACGAGTACAAGGTGCCCAGCAAGAAATTCAAGGTGCTGG<br>GCAACACCGACCGGCACAGCATCAAGAAGAACCTGATCGGAGCC<br>CTGCTGTTCGACAGCGGCGAAACAGCCGAGGCCACCCGGCTGAA<br>GAGAACCGCCAGAAGAAGATACACCAGACGGAAGAACCGGATCT<br>GCTATCTGCAAGAGATCTTCAGCAACGAGATGGCCAAGGTGGACG<br>ACAGCTTCTTCCACAGACTGGAAGAGTCCTTCCTGGTGGAAGAGG<br>ATAAGAAGCACGAGCGGCACCCCATCTTCGGCAACATCGTGGAC<br>GAGGTGGCCTACCACGAGAAGTACCCCACCATCTACCACCTGAGA<br>AAGAAACTGGTGGACAGCACCGACAAGGCCGACCTGCGGCTGAT<br>CTATCTGGCCCTGGCCCACATGATCAAGTTCCGGGGCCACTTCCT<br>GATCGAGGGCGACCTGAACCCCGACAACAGCGACGTGGACAAGC<br>TGTTCATCCAGCTGGTGCAGACCTACAACCAGCTGTTCGAGGAAA<br>ACCCCATCAACGCCAGCGGCGTGGACGCCAAGGCCATCCTGTCT<br>GCCAGACTGAGCAAGAGCAGACGGCTGGAAAATCTGATCGCCCA<br>GCTGCCCGGCGAGAAGAAGAATGGCCTGTTCGGAAACCTGATTG<br>CCCTGAGCCTGGGCCTGACCCCCAACTTCAAGAGCAACTTCGACC<br>TGGCCGAGGATGCCAAACTGCAGCTGAGCAAGGACACCTACGAC<br>GACGACCTGGACAACCTGCTGGCCCAGATCGGCGACCAGTACGC<br>CGACCTGTTTCTGGCCGCCAAGAACCTGTCCGACGCCATCCTGCT<br>GAGCGACATCCTGAGAGTGAACACCGAGATCACCAAGGCCCCCC<br>TGAGCGCCTCTATGATCAAGAGATACGACGAGCACCACCAGGACC<br>TGACCCTGCTGAAAGCTCTCGTGCGGCAGCAGCTGCCTGAGAAG<br>TACAAAGAGATTTTCTTCGACCAGAGCAAGAACGGCTACGCCGGC<br>TACATTGACGGCGGAGCCAGCCAGGAAGAGTTCTACAAGTTCATC

No.	Name	Purpose	Generation strategy
			AAGCCCATCCTGGAAAAGATGGACGGCACCGAGGAACTGCTCGT<br>GAAGCTGAACAGAGAGGACCTGCTGCGGAAGCAGCGGACCTTCG<br>ACAACGGCAGCATCCCCCACCAGATCCACCTGGGAGAGCTGCAC<br>GCCATTCTGCGGCGGCAGGAAGATTTTTACCCATTCCTGAAGGAC<br>AACCGGGAAAAGATCGAGAAGATCCTGACCTTCCGCATCCCCTAC<br>TACGTGGGCCCTCTGGCCAGGGGAAACAGCAGATTCGCCTGGAT<br>GACCAGAAAGAGCGAGGAAACCATCACCCCCTGGAACTTCGAGG<br>AAGTGGTGGACAAGGGCGCTTCCGCCCAGAGCTTCATCGAGCGG<br>ATGACCAACTTCGATAAGAACCTGCCCAACGAGAAGGTGCTGCCC<br>AAGCACAGCCTGCTGTACGAGTACTTCACCGTGTATAACGAGCTG<br>ACCAAAGTGAAATACGTGACCGAGGGAATGAGAAAGCCCGCCTTC<br>CTGAGCGGCGAGCAGAAAAAGGCCATCGTGGACCTGCTGTTCAA<br>GACCAACCGGAAAGTGACCGTGAAGCAGCTGAAAGAGGACTACTT<br>CAAGAAAATCGAGTGCTTCGACTCCGTGGAAATCTCCGGCGTGGA<br>AGATCGGTTCAACGCCTCCCTGGGCACATACCACGATCTGCTGAA<br>AATTATCAAGGACAAGGACTTCCTGGACAATGAGGAAAACGAGGA<br>CATTCTGGAAGATATCGTGCTGACCCTGACACTGTTTGAGGACAG<br>AGAGATGATCGAGGAACGGCTGAAAACCTATGCCCACCTGTTCGA<br>CGACAAAGTGATGAAGCAGCTGAAGCGGCGGAGATACACCGGCT<br>GGGGCAGGCTGAGCCGGAAGCTGATCAACGGCATCCGGGACAA<br>GCAGTCCGGCAAGACAATCCTGGATTTCCTGAAGTCCGACGGCTT<br>CGCCAACAGAAACTTCATGCAGCTGATCCACGACGACAGCCTGAC<br>CTTTAAAGAGGACATCCAGAAAGCCCAGGTGTCCGGCCAGGGCG<br>ATAGCCTGCACGAGCACATTGCCAATCTGGCCGGCAGCCCCGCC<br>ATTAAGAAGGGCATCCTGCAGACAGTGAAGGTGGTGGACGAGCT<br>CGTGAAAGTGATGGGCCGGCACAAGCCCGAGAACATCGTGATCG<br>AAATGGCCAGAGAGAACCAGACCACCCAGAAGGGACAGAAGAAC<br>AGCCGCGAGAGAATGAAGCGGATCGAAGAGGGCATCAAAGAGCT<br>GGGCAGCCAGATCCTGAAAGAACACCCCGTGGAAAACACCCAGC<br>TGCAGAACGAGAAGCTGTACCTGTACTACCTGCAGAATGGGCGG<br>GATATGTACGTGGACCAGGAACTGGACATCAACCGGCTGTCCGAC<br>TACGATGTGGACCATATCGTGCCTCAGAGCTTTCTGAAGGACGAC<br>TCCATCGACAACAAGGTGCTGACCAGAAGCGACAAGAACCGGGG<br>CAAGAGCGACAACGTGCCCTCCGAAGAGGTCGTGAAGAAGATGA<br>AGAACTACTGGCGGCAGCTGCTGAACGCCAAGCTGATTACCCAGA<br>GAAAGTTCGACAATCTGACCAAGGCCGAGAGAGGCGGCCTGAGC<br>GAACTGGATAAGGCCGGCTTCATCAAGAGACAGCTGGTGGAAAC<br>CCGGCAGATCACAAAGCACGTGGCACAGATCCTGGACTCCCGGA<br>TGAACACTAAGTACGACGAGAATGACAAGCTGATCCGGGAAGTGA<br>AAGTGATCACCCTGAAGTCCAAGCTGGTGTCCGATTTCCGGAAGG<br>ATTTCCAGTTTTACAAAGTGCGCGAGATCAACAACTACCACCACGC<br>CCACGACGCCTACCTGAACGCCGTCGTGGGAACCGCCCTGATCA<br>AAAAGTACCCTAAGCTGGAAAGCGAGTTCGTGTACGGCGACTACA<br>AGGTGTACGACGTGCGGAAGATGATCGCCAAGAGCGAGCAGGAA<br>ATCGGCAAGGCTACCGCCAAGTACTTCTTCTACAGCAACATCATG<br>AACTTTTTCAAGACCGAGATTACCCTGGCCAACGGCGAGATCCGG<br>AAGCGGCCTCTGATCGAGACAAACGGCGAAACCGGGGAGATCGT<br>GTGGGATAAGGGCCGGGATTTTGCCACCGTGCGGAAAGTGCTGA<br>GCATGCCCCAAGTGAATATCGTGAAAAAGACCGAGGTGCAGACAG<br>GCGGCTTCAGCAAAGAGTCTATCCTGCCCAAGAGGAACAGCGATA<br>AGCTGATCGCCAGAAAGAAGGACTGGGACCCTAAGAAGTACGGC<br>GGCTTCGACAGCCCCACCGTGGCCTATTCTGTGCTGGTGGTGGC<br>CAAAGTGGAAAAGGGCAAGTCCAAGAAACTGAAGAGTGTGAAAGA<br>GCTGCTGGGGATCACCATCATGGAAAGAAGCAGCTTCGAGAAGAA<br>TCCCATCGACTTTCTGGAAGCCAAGGGCTACAAAGAAGTGAAAAA<br>GGACCTGATCATCAAGCTGCCTAAGTACTCCCTGTTCGAGCTGGA<br>AAACGGCCGGAAGAGAATGCTGGCCTCTGCCGGCGAACTGCAGA<br>AGGGAAACGAACTGGCCCTGCCCTCCAAATATGTGAACTTCCTGT<br>ACCTGGCCAGCCACTATGAGAAGCTGAAGGGCTCCCCCGAGGAT<br>AATGAGCAGAAACAGCTGTTTGTGGAACAGCACAAGCACTACCTG<br>GACGAGATCATCGAGCAGATCAGCGAGTTCTCCAAGAGAGTGATC<br>CTGGCCGACGCTAATCTGGACAAAGTGCTGTCCGCCTACAACAAG<br>CACCGGGATAAGCCCATCAGAGAGCAGGCCGAGAATATCATCCA<br>CCTGTTTACCCTGACCAATCTGGGAGCCCCTGCCGCCTTCAAGTA<br>CTTTGACACCACCATCGACCGGAAGAGGTACACCAGCACCAAAGA<br>GGTGCTGGACGCCACCCTGATCCACCAGAGCATCACCGGCCTGT<br>ACGAGACACGGATCGACCTGTCTCAGCTGGGAGGCGACAAAAGG<br>CCGGCGGCCACGAAAAAGGCCGGccaggcaaaaaagaaaaaggGATCC

No.	Name	Purpose	Generation strategy
			TCTGAGACACCAGGCACCTCCGAGTCTGCCACCCCTGAGGGAGG<br>AGCACCAGAGGTGACAGCCACCGTGATCTCCTACGATAACTATGT<br>GACAATCCTGGACGAGGAGACACTGAAGGCCTGGATCGCCAAGC<br>TGGAGAAGGCCCCCGTGTTCGCCTTTGATACAGAGACAGACAGCC<br>TGGATAACATCTCCGCCAATCTGGTGGGCCTGTCTTTCGCCATCG<br>AGCCTGGCGTGGCCGCCTATATCCCAGTGGCCCACGACTACCTG<br>GATGCCCCCGACCAGATCAGCAGGGAGAGAGCCCTGGAGCTGCT<br>GAAGCCTCTGCTGGAGGATGAGAAGGCCCTGAAGGTCGGCCAGA<br>ACCTGAAGTATGACAGGGGCATCCTGGCCAATTACGGCATCGAGC<br>TGAGAGGCATCGCCTTTGACACCATGCTGGAGTCTTATATCCTGA<br>ATAGCGTGGCAGGCCGGCACGACATGGATTCCCTGGCCGAGAGG<br>TGGCTGAAGCACAAGACAATCACCTTCGAGGAGATCGCCGGCAA<br>GGGCAAGAACCAGCTGACCTTCAACCAGATCGCCCTGGAGGAGG<br>CAGGCAGGTACGCAGCAGAGGACGCAGATGTGACCCTGCAGCTG<br>CACCTGAAGATGTGGCCAGATCTGCAGAAGCACtag. Peptide sequence:<br>MDYKDHDGDYKDHDIDYKDDDDKMAPKKKRKVGIHGVPAADKKYSI<br>GLDIGTNSVGWAVITDEYKVPSKKFKVLGNTDRHSIKKNLIGALLFDS<br>GETAEATRLKRTARRRYTRRKNRICYLQEIFSNEMAKVDDSFFHRLE<br>ESFLVEEDKKHERHPIFGNIVDEVAYHEKYPTIYHLRKKLVDSTDKADL<br>RLIYLALAHMIKFRGHFLIEGDLNPDNSDVDKLFIQLVQTYNQLFEENPI<br>NASGVDAKAILSARLSKSRRLENLIAQLPGEKKNGLFGNLIALSLGLTP<br>NFKSNFDLAEDAKLQLSKDTYDDDLDNLLAQIGDQYADLFLAAKNLSD<br>AILLSDILRVNTEITKAPLSASMIKRYDEHHQDLTLLKALVRQQLPEKYK<br>EIFFDQSKNGYAGYIDGGASQEEFYKFIKPILEKMDGTEELLVKLNRE<br>DLLRKQRTFDNGSIPHQIHLGELHAILRRQEDFYPFLKDNREKIEKILTF<br>RIPYYVGPLARGNSRFAWMTRKSEETITPWNFEEVVDKGASAQSFIE<br>RMTNFDKNLPNEKVLPKHSLLYEYFTVYNELTKVKYVTEGMRKPAFL<br>SGEQKKAIVDLLFKTNRKVTVKQLKEDYFKKIECFDSVEISGVEDRFN<br>ASLGTYHDLLKIIKDKDFLDNEENEDILEDIVLTLTLFEDREMIEERLKTY<br>AHLFDDKVMKQLKRRRYTGWGRLSRKLINGIRDKQSGKTILDFLKSD<br>GFANRNFMQLIHDDSLTFKEDIQKAQVSGQGDSLHEHIANLAGSPAIK<br>KGILQTVKVVDELVKVMGRHKPENIVIEMARENQTTQKGQKNSRERM<br>KRIEEGIKELGSQILKEHPVENTQLQNEKLYLYYLQNGRDMYVDQELD<br>INRLSDYDVDHIVPQSFLKDDSIDNKVLTRSDKNRGKSDNVPSEEVVK<br>KMKNYWRQLLNAKLITQRKFDNLTKAERGGLSELDKAGFIKRQLVET<br>RQITKHVAQILDSRMNTKYDENDKLIREVKVITLKSKLVSDFRKDFQFY<br>KVREINNYHHAHDAYLNAVVGTALIKKYPKLESEFVYGDYKVYDVRK<br>MIAKSEQEIGKATAKYFFYSNIMNFFKTEITLANGEIRKRPLIETNGETG<br>EIVWDKGRDFATVRKVLSMPQVNIVKKTEVQTGGFSKESILPKRNSD<br>KLIARKKDWDPKKYGGFDSPTVAYSVLVVAKVEKGKSKKLKSVKELL<br>GITIMERSSFEKNPIDFLEAKGYKEVKKDLIIKLPKYSLFELENGRKRML<br>ASAGELQKGNELALPSKYVNFLYLASHYEKLKGSPEDNEQKQLFVEQ<br>HKHYLDEIIEQISEFSKRVILADANLDKVLSAYNKHRDKPIREQAENIIHL<br>FTLTNLGAPAAFKYFDTTIDRKRYTSTKEVLDATLIHQSITGLYETRIDL<br>SQLGGDKRPAATKKAGQAKKKKGSSETPGTSESATPEGGAPEVTAT<br>VISYDNYVTILDEETLKAWIAKLEKAPVFAFDTETDSLDNISANLVGLSF<br>AIEPGVAAYIPVAHDYLDAPDQISRERALELLKPLLEDEKALKVGQNLK<br>YDRGILANYGIELRGIAFDTMLESYILNSVAGRHDMDSLAERWLKHKTI<br>TFEEIAGKGKNQLTFNQIALEEAGRYAAEDADVTLQLHLKMWPDLQK<br>H(Color Key: Cas9-linker-3' exo)
5	pspCas9-Exo-tetra-com-hCLCN5-sp-g1	Plasmid for expressing a fusion protein of spCas9 and the 5' and 3' exonuclease domain of POLI, and sgRNA targeting human CLCN5 gene.	Cut pspCas9-POLI-tetra-com-hCLCN5-sp-g1 with AccI+NheI to remove the DNA coding for the DNA polymerase activity. Treat the DNA with the Klenow fragment of DNA POLI to make blunt ends. Then use T4 DNA ligase to ligate the ends.<br>Nucleic acid sequence:<br>ATGGACTATAAGGACCACGACGGAGACTACAAGGATCATGATATT<br>GATTACAAAGACGATGACGATAAGATGGCCCCAAAGAAGAAGCGG<br>AAGGTCGGTATCCACGGAGTCCCAGCAGCCGACAAGAAGTACAG<br>CATCGGCCTGGACATCGGCACCAACTCTGTGGGCTGGGCCGTGA<br>TCACCGACGAGTACAAGGTGCCCAGCAAGAAATTCAAGGTGCTGG<br>GCAACACCGACCGGCACAGCATCAAGAAGAACCTGATCGGAGCC<br>CTGCTGTTCGACAGCGGCGAAACAGCCGAGGCCACCCGGCTGAA<br>GAGAACCGCCAGAAGAAGATACACCAGACGGAAGAACCGGATCT<br>GCTATCTGCAAGAGATCTTCAGCAACGAGATGGCCAAGGTGGACG<br>ACAGCTTCTTCCACAGACTGGAAGAGTCCTTCCTGGTGGAAGAGG<br>ATAAGAAGCACGAGCGGCACCCCATCTTCGGCAACATCGTGGAC<br>GAGGTGGCCTACCACGAGAAGTACCCCACCATCTACCACCTGAGA

No.	Name	Purpose	Generation strategy
			AAGAAACTGGTGGACAGCACCGACAAGGCCGACCTGCGGCTGAT<br>CTATCTGGCCCTGGCCCACATGATCAAGTTCCGGGGCCACTTCCT<br>GATCGAGGGCGACCTGAACCCCGACAACAGCGACGTGGACAAGC<br>TGTTCATCCAGCTGGTGCAGACCTACAACCAGCTGTTCGAGGAAA<br>ACCCCATCAACGCCAGCGGCGTGGACGCCAAGGCCATCCTGTCT<br>GCCAGACTGAGCAAGAGCAGACGGCTGGAAAATCTGATCGCCCA<br>GCTGCCCGGCGAGAAGAAGAATGGCCTGTTCGGAAACCTGATTG<br>CCCTGAGCCTGGGCCTGACCCCCAACTTCAAGAGCAACTTCGACC<br>TGGCCGAGGATGCCAAACTGCAGCTGAGCAAGGACACCTACGAC<br>GACGACCTGGACAACCTGCTGGCCCAGATCGGCGACCAGTACGC<br>CGACCTGTTTCTGGCCGCCAAGAACCTGTCCGACGCCATCCTGCT<br>GAGCGACATCCTGAGAGTGAACACCGAGATCACCAAGGCCCCCC<br>TGAGCGCCTCTATGATCAAGAGATACGACGAGCACCACCAGGACC<br>TGACCCTGCTGAAAGCTCTCGTGCGGCAGCAGCTGCCTGAGAAG<br>TACAAAGAGATTTTCTTCGACCAGAGCAAGAACGGCTACGCCGGC<br>TACATTGACGGCGGAGCCAGCCAGGAAGAGTTCTACAAGTTCATC<br>AAGCCCATCCTGGAAAAGATGGACGGCACCGAGGAACTGCTCGT<br>GAAGCTGAACAGAGAGGACCTGCTGCGGAAGCAGCGGACCTTCG<br>ACAACGGCAGCATCCCCCACCAGATCCACCTGGGAGAGCTGCAC<br>GCCATTCTGCGGCGGCAGGAAGATTTTTACCCATTCCTGAAGGAC<br>AACCGGGAAAAGATCGAGAAGATCCTGACCTTCCGCATCCCCTAC<br>TACGTGGGCCCTCTGGCCAGGGGAAACAGCAGATTCGCCTGGAT<br>GACCAGAAAGAGCGAGGAAACCATCACCCCCTGGAACTTCGAGG<br>AAGTGGTGGACAAGGGCGCTTCCGCCCAGAGCTTCATCGAGCGG<br>ATGACCAACTTCGATAAGAACCTGCCCAACGAGAAGGTGCTGCCC<br>AAGCACAGCCTGCTGTACGAGTACTTCACCGTGTATAACGAGCTG<br>ACCAAAGTGAAATACGTGACCGAGGGAATGAGAAAGCCCGCCTTC<br>CTGAGCGGCGAGCAGAAAAAGGCCATCGTGGACCTGCTGTTCAA<br>GACCAACCGGAAAGTGACCGTGAAGCAGCTGAAAGAGGACTACTT<br>CAAGAAAATCGAGTGCTTCGACTCCGTGGAAATCTCCGGCGTGGA<br>AGATCGGTTCAACGCCTCCCTGGGCACATACCACGATCTGCTGAA<br>AATTATCAAGGACAAGGACTTCCTGGACAATGAGGAAAACGAGGA<br>CATTCTGGAAGATATCGTGCTGACCCTGACACTGTTTGAGGACAG<br>AGAGATGATCGAGGAACGGCTGAAAACCTATGCCCACCTGTTCGA<br>CGACAAAGTGATGAAGCAGCTGAAGCGGCGGAGATACACCGGCT<br>GGGGCAGGCTGAGCCGGAAGCTGATCAACGGCATCCGGGACAA<br>GCAGTCCGGCAAGACAATCCTGGATTTCCTGAAGTCCGACGGCTT<br>CGCCAACAGAAACTTCATGCAGCTGATCCACGACGACAGCCTGAC<br>CTTTAAAGAGGACATCCAGAAAGCCCAGGTGTCCGGCCAGGGCG<br>ATAGCCTGCACGAGCACATTGCCAATCTGGCCGGCAGCCCCGCC<br>ATTAAGAAGGGCATCCTGCAGACAGTGAAGGTGGTGGACGAGCT<br>CGTGAAAGTGATGGGCCGGCACAAGCCCGAGAACATCGTGATCG<br>AAATGGCCAGAGAGAACCAGACCACCCAGAAGGGACAGAAGAAC<br>AGCCGCGAGAGAATGAAGCGGATCGAAGAGGGCATCAAAGAGCT<br>GGGCAGCCAGATCCTGAAAGAACACCCCGTGGAAAACACCCAGC<br>TGCAGAACGAGAAGCTGTACCTGTACTACCTGCAGAATGGGCGG<br>GATATGTACGTGGACCAGGAACTGGACATCAACCGGCTGTCCGAC<br>TACGATGTGGACCATATCGTGCCTCAGAGCTTTCTGAAGGACGAC<br>TCCATCGACAACAAGGTGCTGACCAGAAGCGACAAGAACCGGGG<br>CAAGAGCGACAACGTGCCCTCCGAAGAGGTCGTGAAGAAGATGA<br>AGAACTACTGGCGGCAGCTGCTGAACGCCAAGCTGATTACCCAGA<br>GAAAGTTCGACAATCTGACCAAGGCCGAGAGAGGCGGCCTGAGC<br>GAACTGGATAAGGCCGGCTTCATCAAGAGACAGCTGGTGGAAAC<br>CCGGCAGATCACAAAGCACGTGGCACAGATCCTGGACTCCCGGA<br>TGAACACTAAGTACGACGAGAATGACAAGCTGATCCGGGAAGTGA<br>AAGTGATCACCCTGAAGTCCAAGCTGGTGTCCGATTTCCGGAAGG<br>ATTTCCAGTTTTACAAAGTGCGCGAGATCAACAACTACCACCACGC<br>CCACGACGCCTACCTGAACGCCGTCGTGGGAACCGCCCTGATCA<br>AAAAGTACCCTAAGCTGGAAAGCGAGTTCGTGTACGGCGACTACA<br>AGGTGTACGACGTGCGGAAGATGATCGCCAAGAGCGAGCAGGAA<br>ATCGGCAAGGCTACCGCCAAGTACTTCTTCTACAGCAACATCATG<br>AACTTTTTCAAGACCGAGATTACCCTGGCCAACGGCGAGATCCGG<br>AAGCGGCCTCTGATCGAGACAAACGGCGAAACCGGGGAGATCGT<br>GTGGGATAAGGGCCGGGATTTTGCCACCGTGCGGAAAGTGCTGA<br>GCATGCCCCAAGTGAATATCGTGAAAAAGACCGAGGTGCAGACAG<br>GCGGCTTCAGCAAAGAGTCTATCCTGCCCAAGAGGAACAGCGATA<br>AGCTGATCGCCAGAAAGAAGGACTGGGACCCTAAGAAGTACGGC<br>GGCTTCGACAGCCCCACCGTGGCCTATTCTGTGCTGGTGGTGGC

No.	Name	Purpose	Generation strategy
			CAAAGTGGAAAAGGGCAAGTCCAAGAAACTGAAGAGTGTGAAAGA<br>GCTGCTGGGGATCACCATCATGGAAAGAAGCAGCTTCGAGAAGAA<br>TCCCATCGACTTTCTGGAAGCCAAGGGCTACAAAGAAGTGAAAAA<br>GGACCTGATCATCAAGCTGCCTAAGTACTCCCTGTTCGAGCTGGA<br>AAACGGCCGGAAGAGAATGCTGGCCTCTGCCGGCGAACTGCAGA<br>AGGGAAACGAACTGGCCCTGCCCTCCAAATATGTGAACTTCCTGT<br>ACCTGGCCAGCCACTATGAGAAGCTGAAGGGCTCCCCCGAGGAT<br>AATGAGCAGAAACAGCTGTTTGTGGAACAGCACAAGCACTACCTG<br>GACGAGATCATCGAGCAGATCAGCGAGTTCTCCAAGAGAGTGATC<br>CTGGCCGACGCTAATCTGGACAAAGTGCTGTCCGCCTACAACAAG<br>CACCGGGATAAGCCCATCAGAGAGCAGGCCGAGAATATCATCCA<br>CCTGTTTACCCTGACCAATCTGGGAGCCCCTGCCGCCTTCAAGTA<br>CTTTGACACCACCATCGACCGGAAGAGGTACACCAGCACCAAAGA<br>GGTGCTGGACGCCACCCTGATCCACCAGAGCATCACCGGCCTGT<br>ACGAGACACGGATCGACCTGTCTCAGCTGGGAGGCGACAAAAGG<br>CCGGCGGCCACGAAAAAGGCCGGccaggcaaaaaagaaaaaggGATCC<br>TCTGAGACACCAGGCACCTCCGAGTCTGCCACCCCTGAGGGAGG<br>AAGCGGAGGAAGCGGCTCCGTGCAGATCCCACAGAACCCCCTGA<br>TCCTGGTGGACGGCAGCTCCTACCTGTATCGGGCCTACCACGCCT<br>TCCCACCTCTGACAAACTCCGCCGGAGAGCCAACCGGAGCCATG<br>TATGGCGTGCTGAATATGCTGAGGAGCCTGATCATGCAGTACAAG<br>CCTACACACGCCGCCGTGGTGTTTGATGCCAAGGGCAAGACCTTC<br>CGCGACGAGCTGTTTGAGCACTACAAGAGCCACAGGCCACCAAT<br>GCCTGACGATCTGAGGGCACAGATCGAGCCACTGCACGCAATGG<br>TGAAGGCCATGGGCCTGCCTCTGCTGGCCGTGAGCGGAGTGGAG<br>GCCGACGATGTGATCGGCACACTGGCAAGGGAGGCAGAGAAGGC<br>AGGCCGCCCAGTGCTGATCTCCACCGGCGACAAGGATATGGCCC<br>AGCTGGTGACACCAAACATCACCCTGATCAACACCATGACAAATA<br>CCATCCTGGGCCCCGAGGAGGTGGTGAATAAGTATGGCGTGCCT<br>CCAGAGCTGATCATCGATTTCCTGGCCCTGATGGGCGACTCTAGC<br>GATAACATCCCTGGCGTGCCAGGAGTGGGAGAAAAGACCGCACA<br>GGCCCTGCTGCAGGGCCTGGGAGGCCTGGACACCCTGTACGCC<br>GAGCCAGAGAAGATCGCCGGCCTGTCCTTTAGGGGCGCCAAGAC<br>AATGGCCGCCAAGCTGGAGCAGAATAAGGAGGTGGCCTACCTGT<br>CTTATCAGCTGGCCACAATCAAGACCGACGTGGAGCTGGAGCTGA<br>CCTGCGAGCAGCTGGAGGTGCAGCAGCCTGCAGCAGAGGAGCTG<br>CTGGGCCTGTTCAAGAAGTACGAGTTTAAGAGATGGACAGCCGAT<br>GTGGAGGCCGGCAAGTGGCTGCAGGCAAAGGGAGCAAAGCCAG<br>CAGCAAAGCCACAGGAGACAAGCGTGGCAGACGAGGCACCAGAG<br>GTGACAGCCACCGTGATCTCCTACGATAACTATGTGACAATCCTG<br>GACGAGGAGACACTGAAGGCCTGGATCGCCAAGCTGGAGAAGGC<br>CCCCGTGTTCGCCTTTGATACAGAGACAGACAGCCTGGATAACAT<br>CTCCGCCAATCTGGTGGGCCTGTCTTTCGCCATCGAGCCTGGCGT<br>GGCCGCCTATATCCCAGTGGCCCACGACTACCTGGATGCCCCCG<br>ACCAGATCAGCAGGGAGAGAGCCCTGGAGCTGCTGAAGCCTCTG<br>CTGGAGGATGAGAAGGCCCTGAAGGTCGGCCAGAACCTGAAGTA<br>TGACAGGGGCATCCTGGCCAATTACGGCATCGAGCTGAGAGGCA<br>TCGCCTTTGACACCATGCTGGAGTCTTATATCCTGAATAGCGTGG<br>CAGGCCGGCACGACATGGATTCCCTGGCCGAGAGGTGGCTGAAG<br>CACAAGACAATCACCTTCGAGGAGATCGCCGGCAAGGGCAAGAA<br>CCAGCTGACCTTCAACCAGATCGCCCTGGAGGAGGCAGGCAGGT<br>ACGCAGCAGAGGACGCAGATGTGACCCTGCAGCTGCACCTGAAG<br>ATGTGGCCAGATCTGCAGAAGCACAAGGGCCCCCTGAACGTCTTT<br>GAGAATATCGAGATGCCCCTGGTGCCTGTGCTGAGCCGGATCGA<br>GCGCAACGGCGTGAAGATCGACCCTAAGGTGCTGCACAATCACTC<br>CGAGGAGCTGACCCTGAGACTGGCCGAGCTGGAGAAGAAGGCCC<br>ACGAGATCGCCGGCGAGGAGTTCAACCTGTCCTCTACAAAGCAGC<br>TGCAGACCATCCTGTTTGAGAAGCAGGGCATCAAGCCCCTGAAGA<br>AAACCCCTGGAGGAGCACCATCTACCAGCGAGGAGGTGCTGGAG<br>GAGCTGGCCCTGGATTATCCCCTGCCTAAAGTGATCCTGGAGTAC<br>CGGGGCCTGGCCAAGCTGAAGTCTctagctcgctttcttgctgtccaatttctattaa<br>aggttcctttgttccctaagtccaactactaa. Peptide sequence:<br>MDYKDHDGDYKDHDIDYKDDDDKMAPKKKRKVGIHGVPAADKKYSI<br>GLDIGTNSVGWAVITDEYKVPSKKFKVLGNTDRHSIKKNLIGALLFDS<br>GETAEATRLKRTARRRYTRRKNRICYLQEIFSNEMAKVDDSFFHRLE<br>ESFLVEEDKKHERHPIFGNIVDEVAYHEKYPTIYHLRKKLVDSTDKADL<br>RLIYLALAHMIKFRGHFLIEGDLNPDNSDVDKLFIQLVQTYNQLFEENPI<br>NASGVDAKAILSARLSKSRRLENLIAQLPGEKKNGLFGNLIALSLGLTP

No.	Name	Purpose	Generation strategy
			NFKSNFDLAEDAKLQLSKDTYDDDLDNLLAQIGDQYADLFLAAKNLSD<br>AILLSDILRVNTEITKAPLSASMIKRYDEHHQDLTLLKALVRQQLPEKYK<br>EIFFDQSKNGYAGYIDGGASQEEFYKFIKPILEKMDGTEELLVKLNRE<br>DLLRKQRTFDNGSIPHQIHLGELHAILRRQEDFYPFLKDNREKIEKILTF<br>RIPYYVGPLARGNSRFAWMTRKSEETITPWNFEEVVDKGASAQSFIE<br>RMTNFDKNLPNEKVLPKHSLLYEYFTVYNELTKVKYVTEGMRKPAFL<br>SGEQKKAIVDLLFKTNRKVTVKQLKEDYFKKIECFDSVEISGVEDRFN<br>ASLGTYHDLLKIIKDKDFLDNEENEDILEDIVLTLTLFEDREMIEERLKTY<br>AHLFDDKVMKQLKRRRYTGWGRLSRKLINGIRDKQSGKTILDFLKSD<br>GFANRNFMQLIHDDSLTFKEDIQKAQVSGQGDSLHEHIANLAGSPAIK<br>KGILQTVKVVDELVKVMGRHKPENIVIEMARENQTTQKGQKNSRERM<br>KRIEEGIKELGSQILKEHPVENTQLQNEKLYLYYLQNGRDMYVDQELD<br>INRLSDYDVDHIVPQSFLKDDSIDNKVLTRSDKNRGKSDNVPSEEVVK<br>KMKNYWRQLLNAKLITQRKFDNLTKAERGGLSELDKAGFIKRQLVET<br>RQITKHVAQILDSRMNTKYDENDKLIREVKVITLKSKLVSDFRKDFQFY<br>KVREINNYHHAHDAYLNAVVGTALIKKYPKLESEFVYGDYKVYDVRK<br>MIAKSEQEIGKATAKYFFYSNIMNFFKTEITLANGEIRKRPLIETNGETG<br>EIVWDKGRDFATVRKVLSMPQVNIVKKTEVQTGGFSKESILPKRNSD<br>KLIARKKDWDPKKYGGFDSPTVAYSVLVVAKVEKGKSKKLKSVKELL<br>GITIMERSSFEKNPIDFLEAKGYKEVKKDLIIKLPKYSLFELENGRKRML<br>ASAGELQKGNELALPSKYVNFLYLASHYEKLKGSPEDNEQKQLFVEQ<br>HKHYLDEIIEQISEFSKRVILADANLDKVLSAYNKHRDKPIREQAENIIHL<br>FTLTNLGAPAAFKYFDTTIDRKRYTSTKEVLDATLIHQSITGLYETRIDL<br>SQLGGDKRPAATKKAGQAKKKKGSSETPGTSESATPEGGSGGSGS<br>VQIPQNPLILVDGSSYLYRAYHAFPPLTNSAGEPTGAMYGVLNMLRSL<br>IMQYKPTHAAVVFDAKGKTFRDELFEHYKSHRPPMPDDLRAQIEPLH<br>AMVKAMGLPLLAVSGVEADDVIGTLAREAEKAGRPVLISTGDKDMAQ<br>LVTPNITLINTMTNTILGPEEVVNKYGVPPELIIDFLALMGDSSDNIPGV<br>PGVGEKTAQALLQGLGGLDTLYAEPEKIAGLSFRGAKTMAAKLEQNK<br>EVAYLSYQLATIKTDVELELTCEQLEVQQPAAEELLGLFKKYEFKRWT<br>ADVEAGKWLQAKGAKPAAKPQETSVADEAPEVTATVISYDNYVTILD<br>EETLKAWIAKLEKAPVFAFDTETDSLDNISANLVGLSFAIEPGVAAYIPV<br>AHDYLDAPDQISRERALELLKPLLEDEKALKVGQNLKYDRGILANYGIE<br>LRGIAFDTMLESYILNSVAGRHDMDSLAERWLKHKTITFEEIAGKGKN<br>QLTFNQIALEEAGRYAAEDADVTLQLHLKMWPDLQKHKGPLNVFENI<br>EMPLVPVLSRIERNGVKIDPKVLHNHSEELTLRLAELEKKAHEIAGEEF<br>NLSSTKQLQTILFEKQGIKPLKKTPGGAPSTSEEVLEELALDYPLPKVIL<br>EYRGLAKLKSLARFLAVQFLLKVPLFPKSNY(Color Key: Cas9-linker-exo)
6	pspCas9-5Exo-tetra-com-hCLCN5-sp-g1	Plasmid for expressing a fusion protein of spCas9 and the 5' exonuclease domain of POLI, and sgRNA targeting human CLCN5 gene.	Cut pspCas9-POLI-tetra-com-hCLCN5-sp-g1 with ClaI+NheI to remove majority of the POLI coding DNA. Then use 5Exo-F (CAGAGCTGATCATCGATTTCCTGG) and 5Exo-R (AagaaagcgagctagccCACGGTGGCTGTCACCTCTGG) to amplify a 400 bp band coding for the 5' exonuclease domain from pspCas9-POLI-tetra-com-hCLCN5-sp-g1. This DNA was inserted into the ClaI+NheI linearized vector by infusion reaction.<br>Nucleic acid sequence:<br>ATGGACTATAAGGACCACGACGGAGACTACAAGGATCATGATATT<br>GATTACAAAGACGATGACGATAAGATGGCCCCAAAGAAGAAGCGG<br>AAGGTCGGTATCCACGGAGTCCCAGCAGCCGACAAGAAGTACAG<br>CATCGGCCTGGACATCGGCACCAACTCTGTGGGCTGGGCCGTGA<br>TCACCGACGAGTACAAGGTGCCCAGCAAGAAATTCAAGGTGCTGG<br>GCAACACCGACCGGCACAGCATCAAGAAGAACCTGATCGGAGCC<br>CTGCTGTTCGACAGCGGCGAAACAGCCGAGGCCACCCGGCTGAA<br>GAGAACCGCCAGAAGAAGATACACCAGACGGAAGAACCGGATCT<br>GCTATCTGCAAGAGATCTTCAGCAACGAGATGGCCAAGGTGGACG<br>ACAGCTTCTTCCACAGACTGGAAGAGTCCTTCCTGGTGGAAGAGG<br>ATAAGAAGCACGAGCGGCACCCCATCTTCGGCAACATCGTGGAC<br>GAGGTGGCCTACCACGAGAAGTACCCCACCATCTACCACCTGAGA<br>AAGAAACTGGTGGACAGCACCGACAAGGCCGACCTGCGGCTGAT<br>CTATCTGGCCCTGGCCCACATGATCAAGTTCCGGGGCCACTTCCT<br>GATCGAGGGCGACCTGAACCCCGACAACAGCGACGTGGACAAGC<br>TGTTCATCCAGCTGGTGCAGACCTACAACCAGCTGTTCGAGGAAA

No.	Name	Purpose	Generation strategy
			ACCCCATCAACGCCAGCGGCGTGGACGCCAAGGCCATCCTGTCT<br>GCCAGACTGAGCAAGAGCAGACGGCTGGAAAATCTGATCGCCCA<br>GCTGCCCGGCGAGAAGAAGAATGGCCTGTTCGGAAACCTGATTG<br>CCCTGAGCCTGGGCCTGACCCCCAACTTCAAGAGCAACTTCGACC<br>TGGCCGAGGATGCCAAACTGCAGCTGAGCAAGGACACCTACGAC<br>GACGACCTGGACAACCTGCTGGCCCAGATCGGCGACCAGTACGC<br>CGACCTGTTTCTGGCCGCCAAGAACCTGTCCGACGCCATCCTGCT<br>GAGCGACATCCTGAGAGTGAACACCGAGATCACCAAGGCCCCCC<br>TGAGCGCCTCTATGATCAAGAGATACGACGAGCACCACCAGGACC<br>TGACCCTGCTGAAAGCTCTCGTGCGGCAGCAGCTGCCTGAGAAG<br>TACAAAGAGATTTTCTTCGACCAGAGCAAGAACGGCTACGCCGGC<br>TACATTGACGGCGGAGCCAGCCAGGAAGAGTTCTACAAGTTCATC<br>AAGCCCATCCTGGAAAAGATGGACGGCACCGAGGAACTGCTCGT<br>GAAGCTGAACAGAGAGGACCTGCTGCGGAAGCAGCGGACCTTCG<br>ACAACGGCAGCATCCCCCACCAGATCCACCTGGGAGAGCTGCAC<br>GCCATTCTGCGGCGGCAGGAAGATTTTTACCCATTCCTGAAGGAC<br>AACCGGGAAAAGATCGAGAAGATCCTGACCTTCCGCATCCCCTAC<br>TACGTGGGCCCTCTGGCCAGGGGAAACAGCAGATTCGCCTGGAT<br>GACCAGAAAGAGCGAGGAAACCATCACCCCCTGGAACTTCGAGG<br>AAGTGGTGGACAAGGGCGCTTCCGCCCAGAGCTTCATCGAGCGG<br>ATGACCAACTTCGATAAGAACCTGCCCAACGAGAAGGTGCTGCCC<br>AAGCACAGCCTGCTGTACGAGTACTTCACCGTGTATAACGAGCTG<br>ACCAAAGTGAAATACGTGACCGAGGGAATGAGAAAGCCCGCCTTC<br>CTGAGCGGCGAGCAGAAAAAGGCCATCGTGGACCTGCTGTTCAA<br>GACCAACCGGAAAGTGACCGTGAAGCAGCTGAAAGAGGACTACTT<br>CAAGAAAATCGAGTGCTTCGACTCCGTGGAAATCTCCGGCGTGGA<br>AGATCGGTTCAACGCCTCCCTGGGCACATACCACGATCTGCTGAA<br>AATTATCAAGGACAAGGACTTCCTGGACAATGAGGAAAACGAGGA<br>CATTCTGGAAGATATCGTGCTGACCCTGACACTGTTTGAGGACAG<br>AGAGATGATCGAGGAACGGCTGAAAACCTATGCCCACCTGTTCGA<br>CGACAAAGTGATGAAGCAGCTGAAGCGGCGGAGATACACCGGCT<br>GGGGCAGGCTGAGCCGGAAGCTGATCAACGGCATCCGGGACAA<br>GCAGTCCGGCAAGACAATCCTGGATTTCCTGAAGTCCGACGGCTT<br>CGCCAACAGAAACTTCATGCAGCTGATCCACGACGACAGCCTGAC<br>CTTTAAAGAGGACATCCAGAAAGCCCAGGTGTCCGGCCAGGGCG<br>ATAGCCTGCACGAGCACATTGCCAATCTGGCCGGCAGCCCCGCC<br>ATTAAGAAGGGCATCCTGCAGACAGTGAAGGTGGTGGACGAGCT<br>CGTGAAAGTGATGGGCCGGCACAAGCCCGAGAACATCGTGATCG<br>AAATGGCCAGAGAGAACCAGACCACCCAGAAGGGACAGAAGAAC<br>AGCCGCGAGAGAATGAAGCGGATCGAAGAGGGCATCAAAGAGCT<br>GGGCAGCCAGATCCTGAAAGAACACCCCGTGGAAAACACCCAGC<br>TGCAGAACGAGAAGCTGTACCTGTACTACCTGCAGAATGGGCGG<br>GATATGTACGTGGACCAGGAACTGGACATCAACCGGCTGTCCGAC<br>TACGATGTGGACCATATCGTGCCTCAGAGCTTTCTGAAGGACGAC<br>TCCATCGACAACAAGGTGCTGACCAGAAGCGACAAGAACCGGGG<br>CAAGAGCGACAACGTGCCCTCCGAAGAGGTCGTGAAGAAGATGA<br>AGAACTACTGGCGGCAGCTGCTGAACGCCAAGCTGATTACCCAGA<br>GAAAGTTCGACAATCTGACCAAGGCCGAGAGAGGCGGCCTGAGC<br>GAACTGGATAAGGCCGGCTTCATCAAGAGACAGCTGGTGGAAAC<br>CCGGCAGATCACAAAGCACGTGGCACAGATCCTGGACTCCCGGA<br>TGAACACTAAGTACGACGAGAATGACAAGCTGATCCGGGAAGTGA<br>AAGTGATCACCCTGAAGTCCAAGCTGGTGTCCGATTTCCGGAAGG<br>ATTTCCAGTTTTACAAAGTGCGCGAGATCAACAACTACCACCACGC<br>CCACGACGCCTACCTGAACGCCGTCGTGGGAACCGCCCTGATCA<br>AAAAGTACCCTAAGCTGGAAAGCGAGTTCGTGTACGGCGACTACA<br>AGGTGTACGACGTGCGGAAGATGATCGCCAAGAGCGAGCAGGAA<br>ATCGGCAAGGCTACCGCCAAGTACTTCTTCTACAGCAACATCATG<br>AACTTTTTCAAGACCGAGATTACCCTGGCCAACGGCGAGATCCGG<br>AAGCGGCCTCTGATCGAGACAAACGGCGAAACCGGGGAGATCGT<br>GTGGGATAAGGGCCGGGATTTTGCCACCGTGCGGAAAGTGCTGA<br>GCATGCCCCAAGTGAATATCGTGAAAAAGACCGAGGTGCAGACAG<br>GCGGCTTCAGCAAAGAGTCTATCCTGCCCAAGAGGAACAGCGATA<br>AGCTGATCGCCAGAAAGAAGGACTGGGACCCTAAGAAGTACGGC<br>GGCTTCGACAGCCCCACCGTGGCCTATTCTGTGCTGGTGGTGGC<br>CAAAGTGGAAAAGGGCAAGTCCAAGAAACTGAAGAGTGTGAAAGA<br>GCTGCTGGGGATCACCATCATGGAAAGAAGCAGCTTCGAGAAGAA<br>TCCCATCGACTTTCTGGAAGCCAAGGGCTACAAAGAAGTGAAAAA<br>GGACCTGATCATCAAGCTGCCTAAGTACTCCCTGTTCGAGCTGGA

No.	Name	Purpose	Generation strategy
			AAACGGCCGGAAGAGAATGCTGGCCTCTGCCGGCGAACTGCAGA<br>AGGGAAACGAACTGGCCCTGCCCTCCAAATATGTGAACTTCCTGT<br>ACCTGGCCAGCCACTATGAGAAGCTGAAGGGCTCCCCCGAGGAT<br>AATGAGCAGAAACAGCTGTTTGTGGAACAGCACAAGCACTACCTG<br>GACGAGATCATCGAGCAGATCAGCGAGTTCTCCAAGAGAGTGATC<br>CTGGCCGACGCTAATCTGGACAAAGTGCTGTCCGCCTACAACAAG<br>CACCGGGATAAGCCCATCAGAGAGCAGGCCGAGAATATCATCCA<br>CCTGTTTACCCTGACCAATCTGGGAGCCCCTGCCGCCTTCAAGTA<br>CTTTGACACCACCATCGACCGGAAGAGGTACACCAGCACCAAAGA<br>GGTGCTGGACGCCACCCTGATCCACCAGAGCATCACCGGCCTGT<br>ACGAGACACGGATCGACCTGTCTCAGCTGGGAGGCGACAAAAGG<br>CCGGCGGCCACGAAAAAGGCCGGccaggcaaaaaagaaaaaggGATCC<br>TCTGAGACACCAGGCACCTCCGAGTCTGCCACCCCTGAGGGAGG<br>AAGCGGAGGAAGCGGCTCCGTGCAGATCCCACAGAACCCCCTGA<br>TCCTGGTGGACGGCAGCTCCTACCTGTATCGGGCCTACCACGCCT<br>TCCCACCTCTGACAAACTCCGCCGGAGAGCCAACCGGAGCCATG<br>TATGGCGTGCTGAATATGCTGAGGAGCCTGATCATGCAGTACAAG<br>CCTACACACGCCGCCGTGGTGTTTGATGCCAAGGGCAAGACCTTC<br>CGCGACGAGCTGTTTGAGCACTACAAGAGCCACAGGCCACCAAT<br>GCCTGACGATCTGAGGGCACAGATCGAGCCACTGCACGCAATGG<br>TGAAGGCCATGGGCCTGCCTCTGCTGGCCGTGAGCGGAGTGGAG<br>GCCGACGATGTGATCGGCACACTGGCAAGGGAGGCAGAGAAGGC<br>AGGCCGCCCAGTGCTGATCTCCACCGGCGACAAGGATATGGCCC<br>AGCTGGTGACACCAAACATCACCCTGATCAACACCATGACAAATA<br>CCATCCTGGGCCCCGAGGAGGTGGTGAATAAGTATGGCGTGCCT<br>CCAGAGCTGATCATCGATTTCCTGGCCCTGATGGGCGACTCTAGC<br>GATAACATCCCTGGCGTGCCAGGAGTGGGAGAAAAGACCGCACA<br>GGCCCTGCTGCAGGGCCTGGGAGGCCTGGACACCCTGTACGCC<br>GAGCCAGAGAAGATCGCCGGCCTGTCCTTTAGGGGCGCCAAGAC<br>AATGGCCGCCAAGCTGGAGCAGAATAAGGAGGTGGCCTACCTGT<br>CTTATCAGCTGGCCACAATCAAGACCGACGTGGAGCTGGAGCTGA<br>CCTGCGAGCAGCTGGAGGTGCAGCAGCCTGCAGCAGAGGAGCTG<br>CTGGGCCTGTTCAAGAAGTACGAGTTTAAGAGATGGACAGCCGAT<br>GTGGAGGCCGGCAAGTGGCTGCAGGCAAAGGGAGCAAAGCCAG<br>CAGCAAAGCCACAGGAGACAAGCGTGGCAGACGAGGCACCAGAG<br>GTGACAGCCACCGTGggctag. Peptide sequence:<br>MDYKDHDGDYKDHDIDYKDDDDKMAPKKKRKVGIHGVPAADKKYSI<br>GLDIGTNSVGWAVITDEYKVPSKKFKVLGNTDRHSIKKNLIGALLFDS<br>GETAEATRLKRTARRRYTRRKNRICYLQEIFSNEMAKVDDSFFHRLE<br>ESFLVEEDKKHERHPIFGNIVDEVAYHEKYPTIYHLRKKLVDSTDKADL<br>RLIYLALAHMIKFRGHFLIEGDLNPDNSDVDKLFIQLVQTYNQLFEENPI<br>NASGVDAKAILSARLSKSRRLENLIAQLPGEKKNGLFGNLIALSLGLTP<br>NFKSNFDLAEDAKLQLSKDTYDDDLDNLLAQIGDQYADLFLAAKNLSD<br>AILLSDILRVNTEITKAPLSASMIKRYDEHHQDLTLLKALVRQQLPEKYK<br>EIFFDQSKNGYAGYIDGGASQEEFYKFIKPILEKMDGTEELLVKLNRE<br>DLLRKQRTFDNGSIPHQIHLGELHAILRRQEDFYPFLKDNREKIEKILTF<br>RIPYYVGPLARGNSRFAWMTRKSEETITPWNFEEVVDKGASAQSFIE<br>RMTNFDKNLPNEKVLPKHSLLYEYFTVYNELTKVKYVTEGMRKPAFL<br>SGEQKKAIVDLLFKTNRKVTVKQLKEDYFKKIECFDSVEISGVEDRFN<br>ASLGTYHDLLKIIKDKDFLDNEENEDILEDIVLTLTLFEDREMIEERLKTY<br>AHLFDDKVMKQLKRRRYTGWGRLSRKLINGIRDKQSGKTILDFLKSD<br>GFANRNFMQLIHDDSLTFKEDIQKAQVSGQGDSLHEHIANLAGSPAIK<br>KGILQTVKVVDELVKVMGRHKPENIVIEMARENQTTQKGQKNSRERM<br>KRIEEGIKELGSQILKEHPVENTQLQNEKLYLYYLQNGRDMYVDQELD<br>INRLSDYDVDHIVPQSFLKDDSIDNKVLTRSDKNRGKSDNVPSEEVVK<br>KMKNYWRQLLNAKLITQRKFDNLTKAERGGLSELDKAGFIKRQLVET<br>RQITKHVAQILDSRMNTKYDENDKLIREVKVITLKSKLVSDFRKDFQFY<br>KVREINNYHHAHDAYLNAVVGTALIKKYPKLESEFVYGDYKVYDVRK<br>MIAKSEQEIGKATAKYFFYSNIMNFFKTEITLANGEIRKRPLIETNGETG<br>EIVWDKGRDFATVRKVLSMPQVNIVKKTEVQTGGFSKESILPKRNSD<br>KLIARKKDWDPKKYGGFDSPTVAYSVLVVAKVEKGKSKKLKSVKELL<br>GITIMERSSFEKNPIDFLEAKGYKEVKKDLIIKLPKYSLFELENGRKRML<br>ASAGELQKGNELALPSKYVNFLYLASHYEKLKGSPEDNEQKQLFVEQ<br>HKHYLDEIIEQISEFSKRVILADANLDKVLSAYNKHRDKPIREQAENIIHL<br>FTLTNLGAPAAFKYFDTTIDRKRYTSTKEVLDATLIHQSITGLYETRIDL<br>SQLGGDKRPAATKKAGQAKKKKGSSETPGTSESATPEGGSGGSGS<br>VQIPQNPLILVDGSSYLYRAYHAFPPLTNSAGEPTGAMYGVLNMLRSL<br>IMQYKPTHAAVVFDAKGKTFRDELFEHYKSHRPPMPDDLRAQIEPLH

No.	Name	Purpose	Generation strategy
			AMVKAMGLPLLAVSGVEADDVIGTLAREAEKAGRPVLISTGDKDMAQLVTPNITLINTMTNTILGPEEVVNKYGVPPELIIDFLALMGDSSDNIPGVPGVGEKTAQALLQGLGGLDTLYAEPEKIAGLSFRGAKTMAAKLEQNKEVAYLSYQLATIKTDVELELTCEQLEVQQPAAEELLGLFKKYEFKRWTADVEAGKWLQAKGAKPAAKPQETSVADEAPEVTATVG (Color Key: Cas9-linker-5' exo)
7	pspCas9-POLI-ST2-com-53-sp-g2	Vector plasmid for expressing spCas9-POLI fusion protein and sgRNA targeting human DMD exon 53.	The AflIII+Acc65I fragment of pspCas9-3'UTR-ST2-com-52-sp-g2 was used to replace that of pspCas9-POLI-tetra-com-hCLCN5-sp-g1 by restriction enzyme digestion and T4 DNA ligase.
8	pspCas9-Klenow-ST2-com-53-sp-g2	Plasmid for expressing spCas9-Klenow fusion protein and sgRNA targeting human DMD exon 53.	The AflIII+Acc65I fragment of pspCas9-3'UTR-ST2-com-52-sp-g2 was used to replace that of pspCas9-Klenow-tetra-com-hCLCN5-sp-g1 by restriction enzyme digestion and T4 DNA ligase.
9	pspCas9-POLI-ST2-com vector	Vector plasmid for expressing spCas9-POLI fusion protein and sgRNA. There are two copies of HBB 3' UTR in the 3'UTR of spCas9-POLI to enhance expression. The ST2 loop of the sgRNA scaffold was replaced by com-aptamer to enable Cas9 RNP encapsulation into viral capsids via com-COM interaction.	The AflIII and Acc65I fragment of pspCas9-3'UTR-ST2-com vector was used to replace the AflIII and Acc65I fragment of pspCas9-POLI-Tetra-com vector by restriction enzyme digestion and T4 DNA ligase.
10	pspCas9-3'UTR-ST2-com-HBB-W	Plasmid for expressing spCas9 and wild type HBB sgRNA. The ST2 loop of the sgRNA scaffold was replaced by com-aptamer to enable Cas9 RNP encapsulation into viral capsids via com-COM interaction.	The annealed HBB-W oligoes were inserted into the BbsI site of pspCas9-3'UTR-ST2-com-vector by T4 DNA ligase.
11	pspCas9-Klenow-ST2-com-vector	Vector plasmid for expressing spCas9-Klenow fusion protein and sgRNA. There are two copies of HBB 3' UTR in the 3'UTR of	The AflIII-Acc65I fragment containing the U6-sgRNA cassette was cut from pspCas9-poli-ST2-com vector and was inserted into the AflIII-Acc65I Sites of pspCas9-Klenow-ST2-com-53-sp-g2 by T4 DNA ligase.

No.	Name	Purpose	Generation strategy
		spCas9-Klenow to enhance expression. The ST2 loop of the sgRNA scaffold was replaced by com-aptamer to enable Cas9 RNP encapsulation into viral capsids via com-Com interaction.	
12	pspCas9-Klenow-ST2-com-HBB-W	Plasmid for expressing spCas9-Klenow fusion protein and sgRNA targeting human HBB.	The annealed HBB oligonucleotides (HBB-sp-WF and HBB-sp-WR) were inserted into the BbsI site of pspCas9-Klenow-ST2-com-vector by T4 DNA ligase.
13	pspCas9-3'UTR-ST2-com-DMD44	Plasmid for expressing spCas9 and sgRNA targeting human DMD exon 44.	The annealed DMD44 oligonucleotides (DMD44-g1-F and DMD44-g1-R) were inserted into the BbsI site of pspCas9-3'UTR-ST2-com-vector by T4 DNA ligase.
14	pspCas9-Klenow-ST2-com-DMD44	Plasmid for expressing spCas9-Klenow fusion protein and sgRNA targeting human DMD exon 44.	The annealed DMD44 oligonucleotides were inserted into the BbsI site of pspCas9-Klenow-ST2-com-vector by T4 DNA ligase.
15	pspCas9-3'UTR-ST2-com-g5	Plasmid for expressing spCas9 and sgRNA targeting human Intragenic 1 (GRCh38.p13, chromosome 20, 32752960–32752979).	Reported in CRISPR J. 2021 Dec;4(6):914-928. doi: 10.1089/crispr.2020.0106. Epub 2021 Mar 16.
16	pspCas9-Klenow-ST2-com-g5	Plasmid for expressing spCas9-Klenow fusion protein and sgRNA targeting Intragenic 1.	The annealed oligonucleotides ABE-g5-F and ABE-g5-R were inserted into the BbsI site of pspCas9-Klenow-ST2-com-vector by T4 DNA ligase.
17	pspCas9-3'UTR-ST2-com-P53	Plasmid for expressing spCas9 and sgRNA targeting human P53.	Reported in CRISPR J. 2021 Dec;4(6):914-928. doi: 10.1089/crispr.2020.0106. Epub 2021 Mar 16.
18	pspCas9-Klenow-ST2-com-P53	Plasmid for expressing spCas9-Klenow fusion protein and sgRNA targeting human P53.	The annealed P53 oligonucleotides (P53-g1F1 and P53-g1R1) were inserted into the BbsI site of pspCas9-Klenow-ST2-com-vector by T4 DNA ligase.

No.	Name	Purpose	Generation strategy
19	pspCas9-3'UTR-ST2-com-MRE11	Plasmid for expressing spCas9 and sgRNA targeting human MRE11.	The annealed MRE11 oligonucleotides (Mre11-gF and Mre11-gR) were inserted into the BbsI site of pspCas9-3'UTR-ST2-com-vector by T4 DNA ligase.
20	pspCas9-Klenow-ST2-com-MRE11	Plasmid for expressing spCas9-Klenow fusion protein and sgRNA targeting human MRE11.	The annealed MRE11 oligonucleotides were inserted into the BbsI site of pspCas9-Klenow-ST2-com-vector by T4 DNA ligase.
21	pspCas9-3'UTR-ST2-com-CtIP	Plasmid for expressing spCas9 and sgRNA targeting human RBBP8.	The annealed CtIP oligonucleotides (CtIP-gF and CtIP-gR) were inserted into the BbsI site of pspCas9-3'UTR-ST2-com-vector by T4 DNA ligase.
22	pspCas9-Klenow-ST2-com-CtIP	Plasmid for expressing spCas9-Klenow fusion protein and sgRNA targeting human TBBP8.	The annealed CtIP oligonucleotides were inserted into the BbsI site of pspCas9-Klenow-ST2-com-vector by T4 DNA ligase.
23	Ppoli-CLCN5-g1	Plasmid for expressing *E. coli* polymerase I with nuclear targeting signals and sgRNA for *CLCN5*.	pspCas9-poli-Tetra-com-clcn5-sp-g1 was cut with PpuMI to delete the 2.6 kb encoding the Cas9 functional domains, and the rest DNA was self-ligated.
24	pspCas9-poli(D705A)-Tetra-com-clcn5-sp-g1	Plasmid for expressing sgRNA for *CLCN5* and Cas9-pol fusion protein with D705A mutation to inactivate the polymerase activity.	Cut pspCas9-poli-Tetra-com-clcn5-sp-g1 with AccI and NheI, and recover the vector (remove the 1 bk band); Use Pol-F1 (GCCAAGCTGAAGTCTACATATAC) and Pol-MR (CTGAGAGTAGGCGGCGGACACG) as primers and use pspCas9-poli-Tetra-com-clcn5-sp-g1 as the template to amplify a fragment of 225 bp (Fragment 1); Use Pol-MF (CGTGTCCGCCGCCTACTCTCAG) and pol-R1 (aagaaagcgagctagcaatgaa) as primers and use pspCas9-poli-Tetra-com-clcn5-sp-g1 as the template to amplify a fragment of 840 bp (Fragment 2). Insert Fragment 1 and 2 into the recovered vector by Infusion reaction.
25	pspCas9-Klenow(D705A)-Tetra-com-clcn5-sp-g1	Plasmid for expressing sgRNA for *CLCN5* and Cas9-Klenow fusion protein with D705A mutation to inactivate the polymerase activity.	The AccI-FseI fragment of pspCas9-poli(D705A)-Tetra-com-clcn5-sp-g1 was replaced by the AccI-FseI fragment of pspCas9-Klenow-Tetra-com-clcn5-sp-g1 to remove the 5' exonuclease coding region by T4 DNA ligase.

Supplementary Table S2. Sequence information for primers and analysis

Primer name	SEQ	Purpose
Reporter-F	tccatttcaggtgtcgtgag	To amplify the DNA in the GFP reporter cassette.
Reporter-R2	TCCAGCTCGACCAGGATG	
HBB-F1	AGCCAGGGCTGGGCATAAAAG	Used to amplify the HBB region for NGS with HBB-R3
HBB-R3	TGGGAAAATAGACCAATAGGCAGAG	Used to amplify the HBB region for NGS with HBB-R or HBB-R
DMD53-F	TCCTGTTGTTCATCATCCTAGC	To amplify the DMD 53 exon target region for NGS
DMD53-R	TCCAGCCATTGTGTTGAATC	
hCLCN5-F	GTTTAAGGGCCCGCCTTTTG	To amplify the CLCN5 target region for NGS
hCLCN5-R	TGTCTTACCTCTCGGTGCCT	
hCLCN5-F3	GACCCAGGTTTCCTGAGCTG	To amplify the CLCN5 target region for SMRT sequencing
hCLCN5-R3	TTCAGAGCTTCCTCCCAAGC	
OT4-F	CCCTATACCTGGGCTCCGTT	To amplify the Off-target 4 region for NGS
OT4-R	GAAAGGGCCTCTCTCTTTGTAATG	
OT5-F	AAGCTCTACAAGGGCAGAGAATG	To amplify the Off-target 8 region for NGS
OT5-R	TCAAAGCTCCCAGATTCACGTT	
OT8-F	CTGCTCTTTGCCTGTTGGAG	To amplify the Off-target 8 region for NGS
OT8-R	GCTAAAGCTGGAAGGCTGTG	
HBD-F2	AAAAGGCAGGGCAGAGTCGACTG	To amplify HBD region for NGS
HBD-R2	GGTAGGAAAACAGCCCAAGGGAC	
DMD44-F	CCATCACCCTTCAGAACCTGA	To amplify DMD exon 44 region for NGS
DMD44-R	tcagtggctaacagaagctga	
CLCN5 5' Sequence	GTAGGATTCTAATCACTGCCTGCTC	Sequences used to find deletions in analyzing SMRT sequencing data
CLCN5 3' Sequence	CTTTCTGCACCTCCTGATAGCCTTG	
MRE11-onF	GCCAAGTGTGAATGTGCACA	To amplify the on-target of for MRE11 NGS
MRE11-onR	CCTCTTAGGCTATGACCAGGG	
Ctip-onF	TCATTGGGAGGCCGAACATC	To amplify the on-target of RBBP8 for NGS
CtIP-onR	AAGGGCTGAAGGATGATGCA	
P53-onF	CTGGCATTCTGGGAGCTTCA	To amplify the on-target of p53 for NGS
P53-onR	GAGACCTGTGGGAAGCGAAA	
g5-onF	GTCTGAGGTCACACAGTGGG	To amplify the on-target of intragenic 1 for NGS
g5-onR	CTGAGAGCAGGGACCACATC	
ABE-g5-F	ACCGGATGAGATAATGATGAGTCA	To make construct for expressing intragenic 1 sgRNA
ABE-g5-R	aaacTGACTCATCATTATCTCATC	
P53-g1F1	ACCGCCATTGTTCAATATCGTCCG	To make construct for expressing *P53* sgRNA
P53-g1R1	AAACCGGACGATATTGAACAATGG	
DMD44-g1-F	CACCGtttagcatgttcccaattctc	To make construct for expressing DMD exon 44 sgRNA
DMD44-g1-R	AAACgagaattgggaacatgctaaaC	
HBB-sp-WF	ACCGGTAACGGCAGACTTCTCCTC	

HBB-sp-WR	AAACGAGGAGAAGTCTGCCGTTAC	To make construct for expressing *HBB* sgRNA
CtlP-gF	accgTTGCCCAAAGATTCCCCAG	To make construct for expressing *RBBP8* sgRNA
CtlP-gR	aaacCTGGGGAATCTTTGGGCAA	
Mre11-gF	accgTGACTGAGATCTGAGTGCTC	To make construct for expressing *MRE11* sgRNA
Mre11-gR	aaacGAGCACTCAGATCTCAGTCA	
53-sp-g2-F	ACCGactgttgcctccggttctga	To make construct for expressing DMD exon 53 sgRNA
53-sp-g2-R	AAACtcagaaccggaggcaacagt	
hCLCN5-sp-g1-F	ACCGGAGGACAAGTCGTACAATGG	To make construct for expressing *CLCN5* sgRNA
hCLCN5-sp-g1-R	AAACCCATTGTACGACTTGTCCTC	

Supplementary Table S3. Single guide RNA target sequences

Name	Protospacer	PAM
CLCN5	GAGGACAAGTCGTACAATGG	TGG
DMD53	ACTGTTGCCTCCGGTTCTGA	AGG
DMD44	TTTAGCATGTTCCCAATTCTC	AGG
HBB	GTAACGGCAGACTTCTCCTC	AGG
Intragenic site 1	GATGAGATAATGATGAGTCA	GGG
P53	CCATTGTTCAATATCGTCCG	GGG
MRE11	TGACTGAGATCTGAGTGCTC	TGG
RBBP8	TTGCCCAAAGATTCCCCAG	GGG

Supplementary Table S4. Overall INDEL rates of co-targeted genes

	RBBP8/CLCN5	
	RBBP8	***CLCN5***
**Cas9**	48.9 ± 1.9	42.4 ± 1.9
**Cas9-Klenow**	1.7 ± 0.03	1.1 ± 0.17

Replicate number is three for all groups. No difference in INDEL rates was observed for co-transfected genes.
